# Cell adhesion heterogeneity reinforces tumour cell dissemination: novel insights from a mathematical model

**DOI:** 10.1186/s13062-017-0188-z

**Published:** 2017-08-11

**Authors:** David Reher, Barbara Klink, Andreas Deutsch, Anja Voss-Böhme

**Affiliations:** 1 Department of Evolutionary Genetics, Max Planck Institute for Evolutionary Anthropology, Deutscher Platz 6, Leipzig, 04103 Germany; 2 Center for Information Services and High Performance Computing, Technische Universität Dresden, Nöthnitzer Str. 46, Dresden, 01062 Germany; 3 Institute for Clinical Genetics, Faculty of Medicine Carl Gustav Carus, Technische Universität Dresden, Fetscherstr. 74, Dresden, 01307 Germany; 4 German Cancer Consortium (DKTK), Dresden, Germany; German Cancer Research Center (DKFZ), Heidelberg, Germany; Center for Molecular Tumor Diagnostics (CMTD), National Center for Tumor Diseases (NCT), Dresden, Germany; 5 Hochschule für Technik und Wirtschaft Dresden, Fakultät Informatik/Mathematik, Friedrich-List-Platz 1, Dresden, 01069 Germany

**Keywords:** Tumour invasion, EMT, Tumour heterogeneity, Metastasis, Mathematical model, Cellular automaton, Intercellular adhesion

## Abstract

**Background:**

Cancer cell invasion, dissemination, and metastasis have been linked to an epithelial-mesenchymal transition (EMT) of individual tumour cells. During EMT, adhesion molecules like E-cadherin are downregulated and the decrease of cell-cell adhesion allows tumour cells to dissociate from the primary tumour mass. This complex process depends on intracellular cues that are subject to genetic and epigenetic variability, as well as extrinsic cues from the local environment resulting in a spatial heterogeneity in the adhesive phenotype of individual tumour cells. Here, we use a novel mathematical model to study how adhesion heterogeneity, influenced by intrinsic and extrinsic factors, affects the dissemination of tumour cells from an epithelial cell population. The model is a multiscale cellular automaton that couples intracellular adhesion receptor regulation with cell-cell adhesion.

**Results:**

Simulations of our mathematical model indicate profound effects of adhesion heterogeneity on tumour cell dissemination. In particular, we show that a large variation of intracellular adhesion receptor concentrations in a cell population reinforces cell dissemination, regardless of extrinsic cues mediated through the local cell density. However, additional control of adhesion receptor concentration through the local cell density, which can be assumed in healthy cells, weakens the effect. Furthermore, we provide evidence that adhesion heterogeneity can explain the remarkable differences in adhesion receptor concentrations of epithelial and mesenchymal phenotypes observed during EMT and might drive early dissemination of tumour cells.

**Conclusions:**

Our results suggest that adhesion heterogeneity may be a universal trigger to reinforce cell dissemination in epithelial cell populations. This effect can be at least partially compensated by a control of adhesion receptor regulation through neighbouring cells. Accordingly, our findings explain how both an increase in intra-tumour adhesion heterogeneity and the loss of control through the local environment can promote tumour cell dissemination.

**Reviewers:**

This article was reviewed by Hanspeter Herzel, Thomas Dandekar and Marek Kimmel.

**Electronic supplementary material:**

The online version of this article (doi:10.1186/s13062-017-0188-z) contains supplementary material, which is available to authorized users.

## Background

The ability of tumours to metastasise is one of the ‘hallmarks of cancer’ [1, 2] and contributes to more than 90% of cancer-related deaths. Before metastases form, tumour cells disseminate and migrate away from solid tumours to invade surrounding tissue [3, 4]. One of the challenges in the development of effective therapies is that the mechanisms that trigger dissemination of single cells or even groups of cells from tumours are not known in great detail [2, 5]. Moreover, several triggers, like hypoxia, may promote cell dissemination in one tumour type and inhibit it in others [6]. A consequence of this is that therapies targeting tumour cell dissemination and metastasis formation are largely under-represented [7]. A better understanding of the general principles underlying the triggers of tumour cell dissemination is therefore a key step in developing effective cancer treatments.

Tumour cell invasion and dissemination are accompanied by epithelial-mesenchymal transitions (EMT) of individual cells [4]. During EMT, epithelial cells gradually and reversibly change their phenotype and adopt a mesenchymal phenotype [8, 9], which exhibits lower adhesion to neighbouring cells, but stronger adhesion to the extracellular matrix compared to the epithelial phenotype [10]. Two of the characteristics of cells undergoing EMT are: an increase in cell motility and downregulation of the epithelial marker E-cadherin [10–15] (also cf. [16] for a comprehensive review on the role of E-cadherin and E-cadherin-related signalling pathways in EMT), the prototypic type-I cadherin which mainly establishes adhesive cell-cell junctions found in epithelial tissues [4]. E-cadherin expression depends on both intrinsic and extrinsic cues. Intrinsic cues relate to effects of intracellular expression regulation, modification, transport and membrane presentation of E-cadherin (cf. [17] for a review of various mechanisms of E-cadherin downregulation and repression in tumours). Genetic mutations in E-cadherin genes lead to differences in E-cadherin expression levels. In particular, there are mutations that can impair the tumour suppressor function of E-cadherin, promote EMT and, therefore, favour tumour cell dissemination [4, 14, 18]. Extrinsic cues refer to influences of the cellular microenvironment. In particular, E-cadherin expression levels increase with local cell density through cadherin-cadherin interactions [19]. This means that the adhesion phenotype - i.e. the expression level of adhesion receptors - of cells is under environmental control as the strength of adhesive cell-cell junctions is correlated with the E-cadherin expression level [20]. Consequently, variations in local cell densities can lead to heterogeneity in E-cadherin expression levels between cells in a tumour.

Intra-tumour heterogeneity can be observed at different levels, e.g. in terms of metabolism and signalling, but also genetically and epigenetically [21, 22]. There is a strong interest to understand the preconditions and implications of tumour heterogeneity [23–25]. Here, we focus on intrinsic and extrinsic preconditions for adhesion heterogeneity and study the implications for tumour cell dissemination with a mathematical model. In particular, we develop a cellular automaton model to account for both the single cell and the cell population level.

Existing mathematical models that analyse effects of cell adhesion on tumour cell dissemination differ by the specific choice of mechanisms included in their respective definitions [26–30] (see also [31] for an overview of models for analysing effects of cell adhesion on cell dissemination). Aspects of adhesion heterogeneity are not considered in any of these models. Nevertheless, there are discrete and hybrid mathematical models in which adhesion heterogeneity is included. For example, Anderson (2005) developed a hybrid model in which cells can have different adhesion phenotypes that do not depend on the cellular microenvironment [32]. Simulations of this model suggest that a decrease of cell-cell adhesion is important in early tumour invasion as it affects tumour cell dissemination. Ramis-Conde et al. (2008) developed an agent-based mathematical model incorporating E-cadherin and *β*-catenin dynamics at the individual cell level [33]. *β*-catenin is an intracellular protein associated with the actin cytoskeleton of a cell. E-cadherins bind to *β*-catenins to form complexes that can interact both with neighbouring cells to form bonds, and with the cytoskeleton of the cell. Computer simulations of this model yield various tumour invasion patterns by a variation of cell-cell adhesive interaction strengths. This model, and an extension by Schlüter et al. (2014) [34], account for adhesion heterogeneity at the individual cell level, but the model is not used to systematically study the effects of intrinsic and extrinsic cues. Domschke et al. (2014) investigated the effect of variability in cell-cell adhesion and adhesion between cells and the extracellular matrix (ECM) on tumour invasion patterns [35]. Their model is based on a system of partial integro-differential equations in which adhesion between cells, and cell-ECM adhesion, are modelled by non-local interaction terms. This study shows that considering dynamic and variable, rather than constant, cell-cell and cell-ECM adhesion parameters can qualitatively describe invasive patterns observed in a number of different types of cancer. The model includes extrinsic cues mediated through the extracellular matrix, but it does not account for intrinsic factors and the local effects of neighbouring cells on adhesion heterogeneity. In summary, even though it is known that spatio-temporal variability in adhesion receptor expression should not be neglected in tumour cell population models [35], the effects of those dynamics have not been studied systematically so far.

We propose a two-dimensional multiscale cellular automaton (CA) model that couples cell-cell adhesion with intracellular adhesion receptor regulation. Cellular automata are widely used as models for different aspects of cancer dynamics [36–43]. A particular type of cellular automata, the lattice-gas cellular automaton (LGCA), is well-suited to model cell-cell interaction and cell migration [28–30]. Several LGCA models have been introduced to study tumour invasion. For instance, Böttger et al. [44–46] and Hatzikirou et al. [47] developed LGCA models to study consequences of the ‘go-or-grow’ dichotomy on cancer growth and invasion. The ‘go-or-grow’ dichotomy is known to characterise glioma cancer cells [48]. Here, we analyse the effects of adhesion heterogeneity on tumour cell dissemination and couple an LGCA model for adhesive cell interaction to an adhesion receptor model adapted from Engwer et al. [49]. For this, we compare simulations of four model scenarios. In particular, we distinguish scenarios in which single cell adhesion receptor regulation is either independent from or controlled by the local cell density and further consider both homogeneous and heterogeneous cell populations, the latter ones with different degrees of adhesion heterogeneity. For our analysis, we count the number of disseminated cells that migrated beyond a threshold distance and measure mean adhesion receptor concentrations of non-disseminated and disseminated cells at a given time. This allows us to characterise potential adhesivity differences between these two subpopulations. We predict that the degree of adhesion heterogeneity determines the size of the disseminated cell subpopulation.

## Methods

### Definition of the multiscale model

We develop a stochastic, spatio-temporal cell-based model to study the effects of cell-cell adhesion heterogeneity on tumour cell dissemination. To do that, a discrete model, namely an individual-based lattice-gas cellular automaton (LGCA) is defined [50, 51]. LGCA are generalisations of probabilistic cellular automata, in which the original concept of binary node states, as often used in statistical physics applications, has been extended to a more complex node structure with so-called velocity channels. This approach, inspired by the FHP-LGCA model for incompressible fluid flow [52], facilitates biological applications. In a biological context, LGCA models are especially well-suited to model cell-cell interaction and cell migration [28–30]. Here, we concentrate on the effects of adhesion heterogeneity on cell dissemination and neglect cell proliferation. In previous studies, we have analysed the effects of cell proliferation on tumour growth and invasion with LGCA models and demonstrated that the particular migration characteristics play an important role in tumour invasion [44, 45, 47]. In our model, individual cells are characterised by their adhesive states, which describe the overall adhesion receptor concentration on the cell surface. The adhesive states are regulated by an adhesion receptor regulation rule which accounts for extrinsic and intrinsic sources of adhesion heterogeneity in the model. Adhesive interactions between cells are realised by a migration rule, which depends on the adhesive states of the cells: cells perform biased random walks such that cells with high adhesive state values have a higher probability to be attracted to neighbouring cells than cells with lower adhesive state values.

**Fig. 1:**
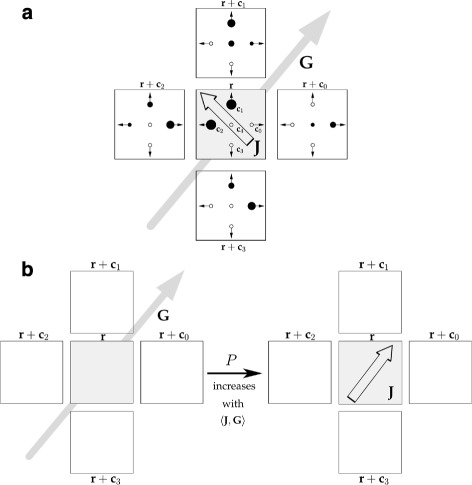
Adhesive cell-cell interaction in the LGCA model. **a** Example configuration of the LGCA; additionally, momentum ***J***(***r***) (*framed arrow*) of the central node and local adhesivity gradient ***G***(***r***) (*gray arrow*) are indicated. State space: Cells are placed on a *square lattice* $\mathcal {L}$ where each node has a substructure with four velocity channels ***c***_*i*_,*i*=0,...,3, and six rest channels (merged into one rest channel in the figure). Accordingly, nodes can host up to ten cells. Adhesive states *a*_*i*_(***r***) of single cells (indicated by *filled dots*) are determined by an adhesion receptor regulation model (see Fig. 2 and Additional file 1 for details). The momentum ***J***(***r***) (*framed arrow*) at a given node ***r*** is the vector sum of all occupation states *η*_*i*_(***r***,*k*), weighted by the adhesive states *a*_*i*_(***r***) (*dot size* symbolises adhesivity strength). The local adhesivity gradient vector ***G***(***r***) (*gray arrow*) at a given node ***r*** is the vector sum of the momenta in the next-neighbour neighbourhood, excluding ***r*** (see Additional file 1). **b** Adhesive interaction is characterised by a reorientation probability *P* that increases with the degree of alignment between local adhesivity gradient ***G***(***r***) (*left*, *gray arrow*) and momentum ***J***(***r***) of the reoriented configuration (*right*, *framed arrow*)

The LGCA model is described on a discrete d-dimensional regular lattice $\mathcal {L}$ with periodic boundary conditions. Each lattice node $\boldsymbol {r} \in \mathcal {L}$ is connected to its *b* nearest neighbours by unit vectors ***c***_*i*_,*i*=0,…,*b*, called velocity channels. The total number of channels per node is defined by *κ*>*b*, where *κ*−*b* is an arbitrary number of channels with zero velocity, called rest channels. Each channel can be occupied by at most one cell at a time, defined by the occupation state variable *η*(***r***,*k*)∈{0,1}. We distinguish moving cells, which reside on the velocity channels, indexed by *i*=1,…,*b*, and resting cells, which are located within the rest channels of the lattice, indexed by *i*=*b*+1,…,*κ*. Adhesive cell states are defined by a variable *a*_*i*_(***r***)∈[0,*∞*). The total number of cells at time *k* and node ***r*** is given by *n*(***r***,*k*). The parameter *κ* is a local cell number bound which is imposed, since the maximal cell number in a given volume is limited in a biological tissue. Notice that *κ* corresponds to a local carrying capacity and thereby prevents cell crowding. Figure 1a illustrates the state space of the LGCA model.

**Fig. 2:**
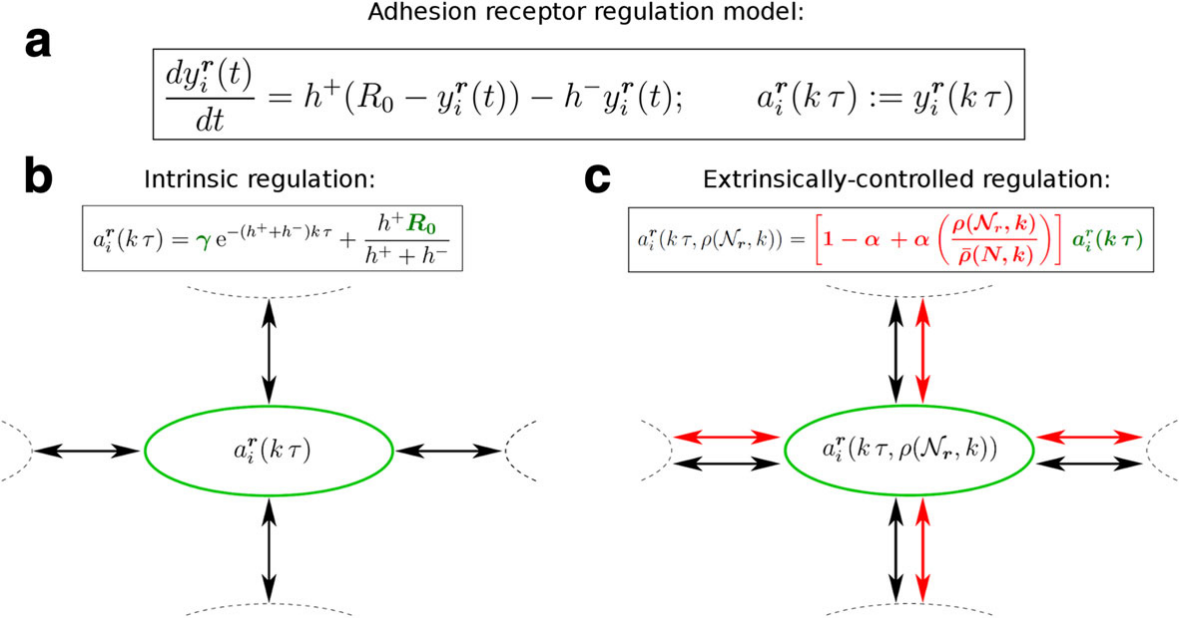
Adhesive state changes of individual cells is modelled by an intracellular adhesion receptor regulation model. **a** Adhesive interactions between cells [*black arrows* in (**b**), (**c**)] are modelled by the probabilistic reorientation operator $\mathcal {R}$ in the LGCA model that depends on adhesive states *a*_*i*_(***r***,*k*) of individual cells determined by the deterministic intracellular adhesion receptor regulation model (Additional file 1). **b** Intrinsic adhesion heterogeneity is modelled by stochastic initial adhesive states *y*_0_ (proportional to *c*, see Additional file 1) and random maximum adhesive states *R*_0_ (*green*). **c** Extrinsically-controlled adhesion heterogeneity is modelled by multiplying adhesive states of single cells by a weight (*red*) that increases linearly with the cell density in the local environment, resulting in an adhesion receptor regulation model that also depends on the occupation states in neighbouring nodes (*red arrows*)

The time evolution of our model is defined by the following rules:

- Adhesion receptor regulation rule: For each cell, located in channel *i* of node ***r***, an updated adhesive state *a* *i*′(***r***) is calculated from the actual adhesive state *a*_*i*_(***r***) by using an ordinary differential equation model described below, which allows to distinguish intrinsic and extrinsic regulation. We assume that cell adhesion receptor expression of single cells can increase with higher local cell densities or that it develops independently from the local environment.
- Migration rule guided by adhesive interaction: Cells perform a biased random walk and move preferentially towards densely populated nodes with high total level of adhesivity. Cells with high adhesive state are particularly sensitive to this rule.

### Adhesion receptor regulation model

#### Deterministic intracellular adhesion receptor regulation model

We describe the adhesion receptor concentration of individual cells positioned at (***r***,***c***_*i*_) at time *k* by an adhesive state variable *a*_*i*_(***r***,*k*). To determine *a*_*i*_(***r***,*k*), we use the solution of an ordinarz differential equation (ODE) adapted from Engwer et al. (2015) [49]:

1 $$ \begin{aligned} \frac{dy_{i}^{\boldsymbol{r}}(t)}{dt}&=h^{+}(R_{0}-y_{i}^{\boldsymbol{r}}(t)) - h^{-}y_{i}^{\boldsymbol{r}}(t), \\ y_{i}^{\boldsymbol{r}}(t)&=c \, \mathrm{e}^{-(h^{+}+h^{-})t}+\frac{h^{+}R_{0}}{h^{+}+h^{-}}, \end{aligned}   $$

in which $y_{i}^{\boldsymbol {r}}(t)$ is the concentration of adhesion receptors on the cell surface at continuous time $t \in \mathbb {R}^{+}_{0}$, $h^{+}, h^{-} \in \mathbb {R}$ are the respective adhesion receptor association and dissociation rates, $R_{0} \in \mathbb {N}$ is the maximum adhesion receptor concentration, and $c \in \mathbb {R}$ is a constant of integration. The initial condition is $y_{i}^{\boldsymbol {r}}(0)=y_{0}$ (see Table 1 for chosen parameter values). The steady state is given by $\frac {h^{+}R_{0}}{h^{+}+h^{-}}$. (see Additional file 1 for further details).

**Table 1:** Parameters for adhesion receptor regulation models

Parameter	Value	Stochastic
*h* ^+^	0.005	no
*h* ^−^	0.005	no
〈*R*_0_〉	100000	yes
〈*y*_0_〉 (*slow regulation mode*)	80000	yes
〈*y*_0_〉 (*fast regulation mode*)	50000	yes

$h^{+}, h^{-} \in \mathbb {R}$ with unit [s^−1^] are the respective adhesion receptor association and dissociation rates, $R_{0} \in \mathbb {N}$ is the maximum adhesion receptor concentration. In the stochastic case, *R*_0_ and *y*_0_ are the expected values of a normal distribution. *y*_0_ has different expected values for *slow* and *fast regulation modes* (see text). Values adapted from Engwer et al. (2015) [49]

We distinguish between a *fast regulation mode*, in which we use a quasi-steady state approximation and assume that the steady state is reached almost instantly, and a *slow regulation mode*. In the latter, we use Eq. (1) to calculate an adhesive state for every discrete cellular automaton time *k* and every cell. The continuous adhesion receptor concentration $y_{i}^{\boldsymbol {r}}(t)$ of a cell at (***r***,***c***_*i*_) is then temporally discretised to give the adhesive state value *a*_*i*_(***r***,*k*) by passing the discrete time-step of the LGCA model to Eq. (1) as an argument [Fig. 2a and Additional file 2 (b)].

#### Heterogeneity in the intracellular adhesion receptor regulation model

We introduce intrinsic adhesion heterogeneity by assigning independent stochastic values to two ODE parameters, the initial adhesive state *y*_0_ and the maximum adhesive state *R*_0_ (Fig. 2). Heterogeneity in these parameters is achieved by randomly selecting values from a normal distribution with fixed expected values for each cell before starting the simulation (Table 1). We use the parameter *γ* to control the degree of heterogeneity (see Additional file 1). The rates *h* ^+^ and *h* ^−^ are held constant and identical for all cells. Note that rates *h* ^+^ and *h* ^−^ have different units compared to rates of second order reactions as $y_{i}^{\boldsymbol {r}}(t)$ is not a molar concentration but the actual number of adhesion receptors on the cell surface [49]. For the *fast regulation mode*, where we approximate Eq. (1) by the steady state value, the parameter *R*_0_, which determines the steady state value $\frac {h^{+}R_{0}}{h^{+}+h^{-}}$, is drawn from a normal distribution with the same parameters as above.

For modelling extrinsic cell density-dependent adhesion receptor regulation, we modify Eq. (1) by considering a linear cell density-dependent weight that is controlled by an environmental control parameter *α*∈[0,1].

### Migration rule guided by adhesive interaction

In the LGCA, the migration rule is implemented in two substeps, (i) a probabilistic reorientation step which redistributes the cells within the velocity channels at a node according to their preferred direction of motion, and (ii) a deterministic propagation step, which moves all cells in velocity channels to their neighbouring nodes in the directions of the velocity channels they reside in.

The probabilistic reorientation step models adhesive interaction as attraction between cells, depending on their adhesive states (see Fig. 1b for an illustration and Additional file 1 for details), and is implemented as a transition probability from a given to a reoriented configuration as follows: a momentum ***J***(***r***) at node ***r*** is defined by weighting the number of cells in a node with their adhesive states *a*_*i*_(***r***). A local adhesivity gradient ***G***(***r***) calculates the vector sum of all momenta of the nearest neighbours of ***r*** (Fig. 1). The reorientation probability *P* is then defined such that it increases with the degree of alignment between the local adhesion gradient ***G***(***r***) and the momentum of the reoriented configuration ***J***(***r***). The detailed mathematical description of the model can be found in Additional file 1.

### Model analysis

**Fig. 3:**
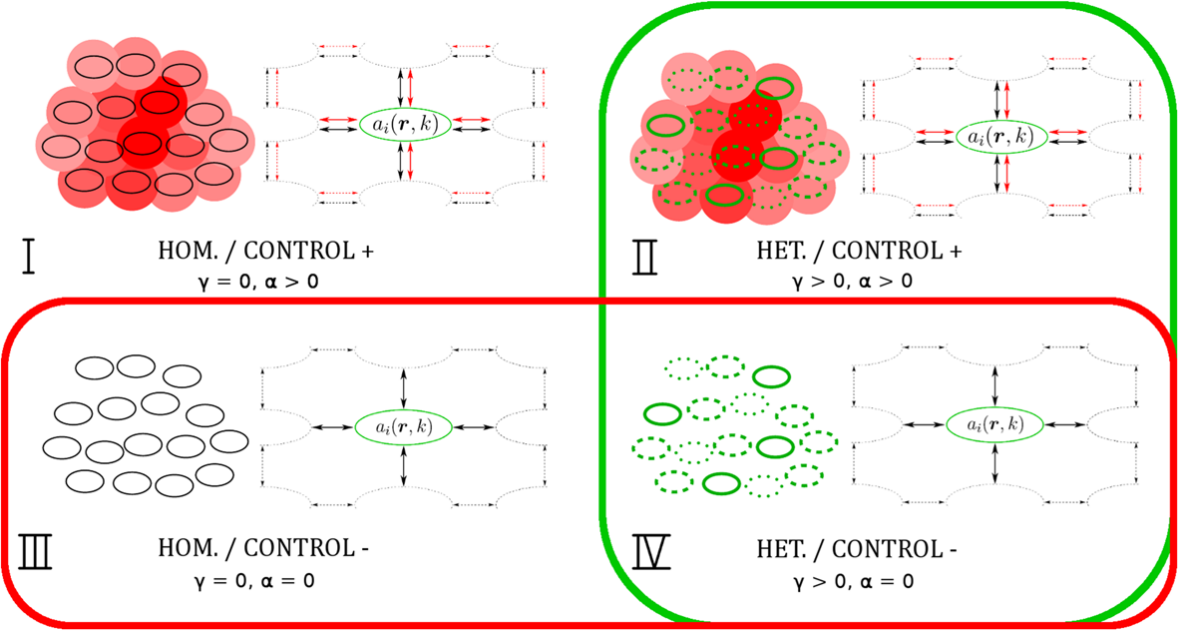
Adhesion receptor regulation scenarios. At the beginning of simulations the tumour cell population of interest can either be homogeneous, i.e. *γ*=0 (HOM), or heterogeneous, i.e. *γ*>0 (HET), regarding intrinsic cell-cell adhesivity. Furthermore, the regulation of single cell adhesion receptor concentration can either be independent, i.e. *α*=0 (CONTROL ^−^), or controlled by the environment, i.e *α*>0 (CONTROL ^+^), the latter via a weight that increases adhesion receptor expression with increasing local cell density. With this weight, we model a cellular adhesion phenotype under environmental control. Combination of these possibilities gives four adhesion receptor regulation scenarios: Scenario I corresponds to a healthy tissue which we assume to be homogeneous and in which adhesion receptor concentration is under environmental control (*γ*=0 and *α*>0). Scenario II corresponds to adhesion heterogeneity caused by differential adhesion receptor expression in the cells, for example due to mutations which are expected to be found in malignant cells (*γ*>0 and *α*>0). Scenario III corresponds to a tissue in which cells are still homogeneous but the environmental control is impaired (*γ*=0 and *α*=0). This is also expected in malignant cells. Scenario IV is a combination of both heterogeneity and impaired environmental control (*γ*>0 and *α*=0)

To investigate the influence of cell adhesion heterogeneity and adhesion receptor regulation on tumour cell dissemination, we study four adhesion receptor regulation scenarios (Fig. 3). First, we distinguish cell populations with intrinsic adhesion heterogeneity (HET), where we model heterogeneity with parameter *γ*>0 as described in the previous section and Additional file 1, and without intrinsic adhesion heterogeneity (HOM, *γ*=0). We use five values for *γ* (0, 0.05, 0.25, 0.4, 0.55) to study different levels of adhesion heterogeneity. Second, we distinguish cell populations in which adhesion receptor regulation of individual cells is density-dependent, i.e. *α*>0 [CONTROL ^+^] or independent, i.e. *α*=0 [CONTROL ^−^].

In summary, in our first scenario, we assume a homogeneous cell population in which adhesion receptor concentration is under environmental control (*γ*=0 and *α*>0) — this corresponds to a healthy tissue. In the second scenario, we introduce adhesion heterogeneity caused by differential adhesion receptor expression in the cells (*γ*>0 and *α*>0). The third scenario corresponds to a homogeneous cell population with impaired environmental control (*γ*=0 and *α*=0). This is also expected in malignant cells, for example due to mutations. The fourth scenario is a combination of both heterogeneous adhesion receptor expression and impaired environmental control (*γ*>0 and *α*=0).

All four regulation scenarios are tested in both a *slow regulation mode* according to Eq. (1) and a *fast regulation mode* in which we use a quasi-steady state approximation. For the latter, we set the adhesion states of all cells to the steady state of the ODE Eq. (1) with standard parameters (Table 1).

In all simulations, we use a circular initial cell configuration mimicking in vitro invasion assays, as described by Justus et al. [53]. The initial configuration comprises both the initial positions as well as the initial adhesive states *y*_0_ of all cells (Table 1). Motivated by a densely packed epithelium, the initial circular cell population has maximum density, i.e. every channel in every node is occupied. We perform all simulations with parameters as in Table 2, averaging results over the number of repetitions. For statistical analysis, we calculate *p*-values from two-tailed t-tests for independent samples with equal variances (F-test, *p*≥0.05) or, in the case of unequal variances (F-test, *p*<0.05) from two-tailed Welch’s t-tests (t _*v*_-test) with Welch-approximated degrees of freedom *v*.

**Table 2:** List of simulation parameters

Simulation parameter	Value
Lattice spacing *ε*	1
⇒ Time-step length *τ*	1
Lattice size	$61 \times 61 \Rightarrow |\mathcal {L}|=3721$
Number of channels *κ*	10, i.e. *β* = 6 (rest channels)
Initial density	Full occupation [1610 cells]
Initial adhesive state (*slow regulation mode*)	50000 [50%, ODE steady state]
Initial adhesive state (*fast regulation mode*)	80000 [80%]
Heterogeneity parameter *γ*	0, 0.05, 0.25, 0.4, 0.55
Environmental control parameter *α*	1
Cell dissemination threshold distance	50
Number of iterations	1000
Number of repetitions	500

These parameters are used for all simulations, unless otherwise noted. Simulations results were averaged over 500 identical repetitions

**Fig. 4:**
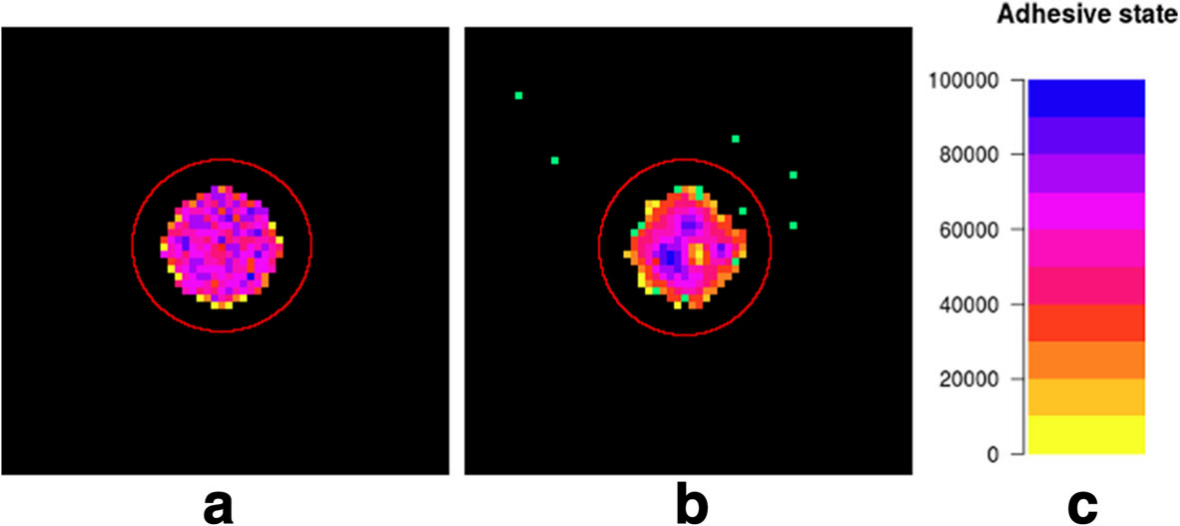
Circular core population and the cell dissemination threshold. **a** Snapshot of an initial configuration and the cell dissemination threshold distance (*red circle*). Nodes without cells are *black*, whereas nodes with occupied channels are *coloured* [*colour bar* legend for adhesive states shown in (**c**)]. The cell population is heterogeneous regarding single cell adhesive states. Note that the mean adhesive states is averaged over all ten channels in a given node. Accordingly, the mean adhesive state at the border nodes of the occupied area is lower due to unoccupied channels. **b** Snapshot of the simulation after 900 time steps. Several cells disseminated from the population and reached the threshold distance indicated by the *red line*. These cells are considered as disseminated cells (*green dots*). Note that disseminated cells are shown in *green* here, independent of their adhesive state

To measure cell dissemination in our model, we define a distance threshold and consider cells as disseminated only if they migrate beyond that distance threshold. We define the cell dissemination threshold distance as the boundary of a ball $B \subset \mathcal {L}$ centred around the initial circular population that is larger than the radius of this initial population (Fig. 4a, *B* corresponds to the area within the red circle): Cells migrating out of *B* are considered as disseminated cells. Invasive cells do not lose their disseminated status once they migrate back into the core cell population (Fig. 4b, green dots).

With the definition of the cell dissemination threshold distance, we can distinguish two subpopulations of cells: non-disseminated cells that stay within the core population and disseminated cells that disseminated from the core cell population and have migrated beyond the cell dissemination threshold distance. The respective cell counts are defined as

2 $$ n^{N} := \sum_{\boldsymbol{r} \in B} n_{\boldsymbol{\eta}(\boldsymbol{r})} \quad \text{and} \quad n^{D} := \sum_{\boldsymbol{r} \in \mathcal{L} \setminus B}n_{\boldsymbol{\eta}(\boldsymbol{r})},   $$

where *n*_***η***(***r***)_ is the number of cells in node ***r*** and superscripts *N* and *D* denote non-disseminated and disseminated cells, respectively.

To analyse the simulations of the four adhesion receptor regulation scenarios (Fig. 3), we introduce three observables: the ratio of disseminated cells, the mean adhesive states of non-disseminated cells, and the adhesivity difference between mean adhesion phenotypes.

#### Ratio of disseminated cells

We count cells that disseminate from the cell population and migrate beyond the cell dissemination threshold distance (Fig. 4). The ratio of the number of disseminated cells to the total cell count Eq. (2) in percent is given by

3 $$ \mathrm{r}_{\text{diss}} := \frac{n^{D}}{n^{D} + n^{N}} \, \, 100\%.   $$

In all simulations, r_diss_ increases with time almost linearly (see Results). We therefore use max(r_diss_), the value of r_diss_ at the end of a simulation, as an observable. To do so, we set r_diss_:= max(r_diss_) and call it the ratio of disseminated cells in the following.

#### Mean adhesive states of non-disseminated and disseminated cells

We are interested in mean adhesive states of non-disseminated cells $\bar {a}^{N}$ and disseminated cells $\bar {a}^{D}$. They are given by

4 $$ \bar{a}^{N} := \frac{1}{n^{N}} \sum_{\boldsymbol{r} \in B} a_{i}(\boldsymbol{r}) \quad \text{and} \quad \bar{a}^{D} := \frac{1}{n^{D}} \sum_{\boldsymbol{r} \in \mathcal{L} \setminus B} a_{i}(\boldsymbol{r}),   $$

respectively. Analogously, we use $\bar {a}$ to describe the mean adhesive state of all cells. For both the *fast* and *slow regulation modes*, the mean adhesive states converge towards a time-invariant equilibrium (Additional file 3). Accordingly, we use the mean adhesive states at the end of a simulation as an approximation for the mean equilibrium adhesive state.

#### Adhesivity difference between mean adhesion phenotypes

To measure the difference between mean adhesion phenotypes, we introduce a distance measure d_*a*_ to give the difference between mean equilibrium adhesive states of non-disseminated $\bar {a}^{N}$ and disseminated cells $\bar {a}^{D}$. Accordingly,

5 $$ \mathrm{d}_{a} := \bar{a}^{N} \, - \, \bar{a}^{D}.   $$

## Results

**The ratio of disseminated cells r**_**diss**_ **increases with increasing adhesion heterogeneity**

**Fig. 5:**
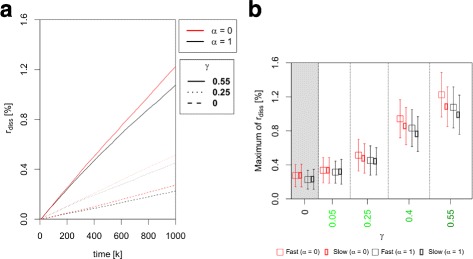
Comparison of the ratio of disseminated cells r_diss_ between simulation scenarios. **a** The plot shows the ratio of disseminated cells [Eq. (3)] over time for all scenarios. The disseminated cell ratio increases linearly over time for all scenarios and *γ*-values. **b** The plot shows the maximum value of the disseminated cell ratio r_diss_ [Eq. (3)] in percent as a function of the adhesion heterogeneity parameter *γ*. The maximum of r_diss_ significantly (*p*<0.001) increases with higher *γ*-values for all scenarios with *γ*>0. For fixed *γ*-values the difference between scenarios III and IV (*α*=0, CONTROL ^−^) and scenarios I and II (*α*=1, CONTROL ^+^) is significant for all *γ* (*p*<0.05). See Additional file 4 for full statistics. Colours of data points are in accordance with scenario colours in Fig. 3

Figure 5a shows that r_diss_, the ratio of the number of disseminated cells to the total cell number, always increases linearly with time. Accordingly, the maximum value of r_diss_ (r_diss_ at *k*=1000) is a suitable observable for comparing the appearance of disseminated cells in the different scenarios. We found an increase of the maximum value of r_diss_ with the adhesion heterogeneity parameter *γ* for all scenarios [Fig. 5b]. This increase is highly significant for all pairwise comparisons of *γ*-values (*p*<0.001, see Additional file 4 for full statistics).

### The ratio of disseminated cells r_diss_ increases in the absence of external adhesion receptor regulation control

**Fig. 6:**
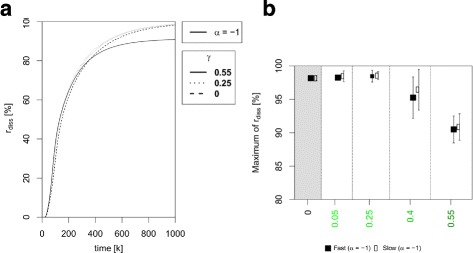
Comparison of the ratio of disseminated cell *r*_diss_ between simulation scenarios with inverted environmental control. In this case adhesive states decrease with increasing local cell density (see Additional file 1). (a) The plot shows the ratio of disseminated cells [Eq. (3)] over time for all scenarios. *r*_diss_-values are strongly increased. (b) The plot shows the maximum value of the disseminated cell ratio *r*_diss_ [Eq. (3)] in percent as a function of the adhesion heterogeneity parameter *γ*. The maximum of *r*_diss_ is higher than 90 *%* for all scenarios. Note the differences in the y-axis compared to Fig. 5

For fixed *γ*-values, the ratio of disseminated cells r_diss_ is always significantly higher for density-independent adhesion receptor regulation [ *α*=0, scenarios III and IV (CONTROL ^−^)] than for the other two scenarios in which cells have density-dependent adhesion receptor regulation (*α*=1, scenarios I and II (CONTROL ^+^), *p*<0.05, see Additional file 5 for full statistics). In contrast, if adhesive states decrease with increasing cell density, the number of disseminated cells strongly increases from less than 2% to more than 90% [Fig. 6b]. However, the increase of r_diss_ with time does not strongly depend on the heterogeneity parameter *γ* in that case [Fig. 6a]. To model this scenario, we changed the density-dependent weighting term such that the term linearly decreases with increasing local cell density (see Additional file 1).

### The adhesion phenotype difference d_*a*_ increases with increasing adhesion heterogeneity

**Fig. 7:**
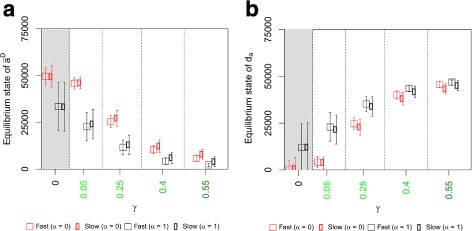
Comparison of adhesion phenotypes between simulation scenarios. **a** The plot shows the mean adhesive state of disseminated cells $\bar {a}^{D}$ [Eq. (4)] in equilibrium (*k*=1000) as a function of the adhesion heterogeneity parameter *γ* for *α*=1). $\bar {a}^{D}$ decreases significantly with higher *γ*-values for all four scenarios (*p*<0.001). For fixed *γ*-values, $\bar {a}^{D}$ is always significantly higher for scenarios III and IV (*α*=0, CONTROL ^−^) than for scenarios I and II (*α*=1, CONTROL ^+^, *p*<0.01). See Additional files 6 and 7 for full statistics. **b** The plot shows the difference between mean adhesion phenotypes in the distance measure d_*a*_ [Eq. (5)] as a function of the adhesion heterogeneity parameter *γ*. Significance levels are similar to (**a**). See Additional files 8 and 9 for full statistics. Colours of data points are in accordance with scenario colours in Fig. 3

The mean adhesive states of both disseminated cells $\bar {a}^{D}$ and non-disseminated cells $\bar {a}^{N}$ reach an equilibrium state for all scenarios for *α*=1 (Additional file 3). This justifies our choice to use the equilibrium values of $\bar {a}^{D}$ and $\bar {a}^{N}$ at *k*=1000 as observables. $\bar {a}^{D}$ decreases with the adhesion heterogeneity parameter *γ* (Fig. 7a, empty symbols). The decrease is highly significant for all pairwise comparisons of *γ*-values for all scenarios with *γ*>0 (*p*<0.001, see Additional file 6 for full statistics). Additionally, we found significantly lower equilibrium $\bar {a}^{D}$-values for density-dependent adhesion receptor regulation [ *α*=1, scenarios I and II (CONTROL ^+^)] than for independent regulation (*α*=0, scenarios III and IV (CONTROL ^−^), *p*<0.05, see Additional file 7 for full statistics). For *γ*≥0.25, the difference is highly significant (*p*<0.001). Additionally, the decrease of the equilibrium $\bar {a}^{D}$-value is significantly higher for the *slow regulation mode* than for the *fast regulation mode* when compared for the same adhesion receptor regulation model (*γ*≥0.25, *p*<0.001). In contrast, $\bar {a}^{N}$ is invariant to changes in *γ* (Additional file 3), resulting in an almost perfect negative correlation between equilibrium values of $\bar {a}^{D}$ and d_*a*_ [Fig. 7b]. Consequently, the equilibrium d_*a*_ increases with the adhesion heterogeneity parameter *γ*. Also resulting from this strong correlation, changes in both $\bar {a}^{D}$ and d_*a*_ as a function of *γ*, but also for fixed *γ*, are of similar significance (see Additional files 6 and 7 for full statistics for $\bar {a}^{D}$ and Additional files 8 and 9 for full statistics for d_*a*_).

**Fig. 8:**
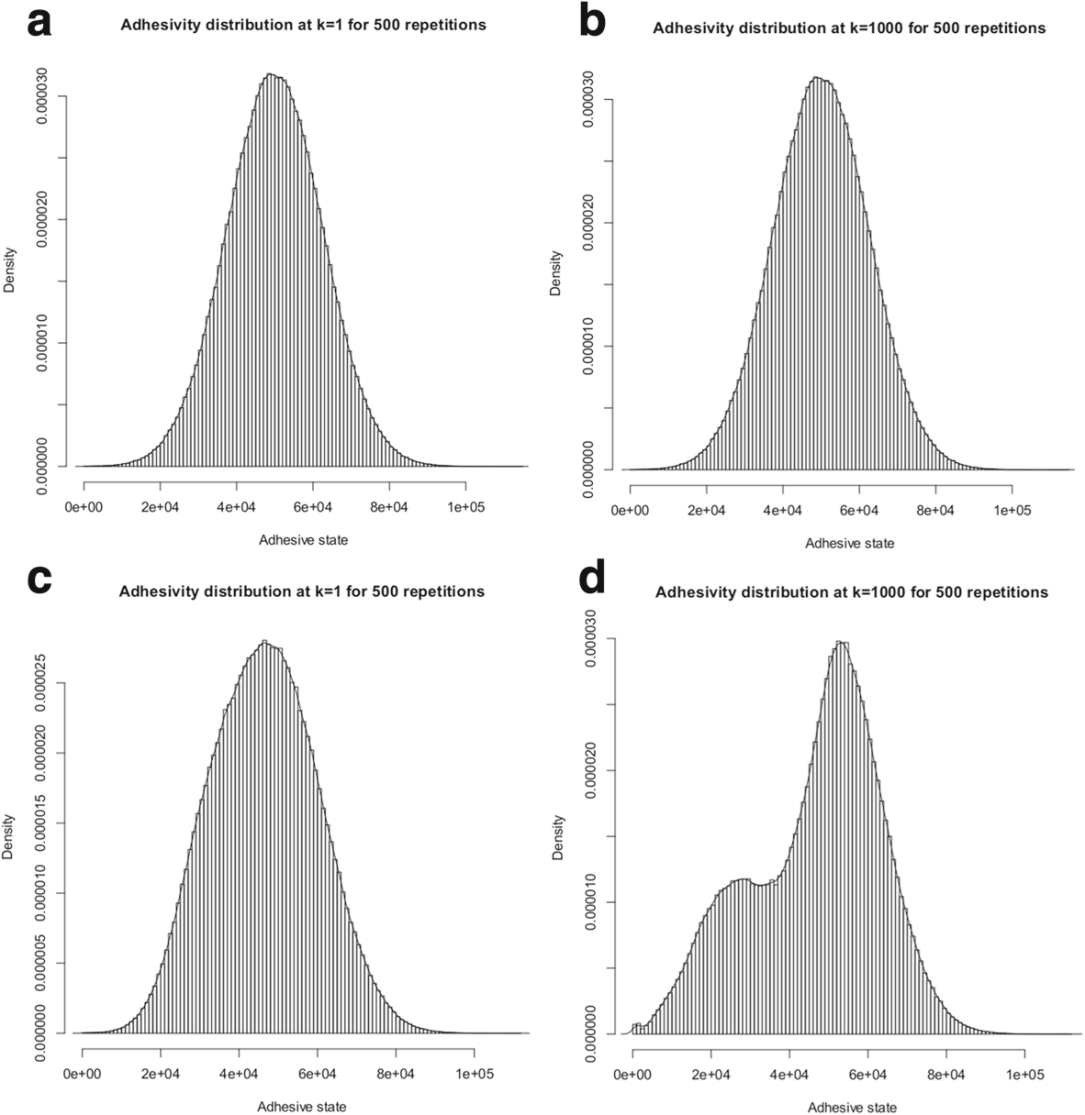
Equilibrium mean adhesive state distributions in cell populations with adhesion heterogeneity (*γ*=0.25) and *fast regulation mode*. **a** and **b** show the distributions of mean adhesive states $\bar {a}$ in equilibrium for cell populations with only intrinsic adhesion heterogeneity (*γ*>0 and *α*=0) and the *fast regulation mode* at time *k*=1 and *k*=1000 (CONTROL ^−^/FAST, Scenario I). There is one expected peak at the steady state of the adhesion receptor regulation model [Eq. (1)]. The distribution stays constant over time. **c** and **d** show the distributions of $\bar {a}$ in equilibrium for cell populations with additional extrinsic adhesion heterogeneity (*γ*>0 and *α*=1) and the *fast regulation mode* at time *k*=1 and *k*=1000 (CONTROL ^+^/FAST, Scenario III). At *k*=1 the distribution is equal to (**a**). At *k*=1000 a second peak at lower adhesive states occurs. The equilibrium adhesivity distributions do not differ when the *slow regulation mode* is considered (Additional file 10)

The increasing difference between the adhesion phenotypes in response to adhesion heterogeneity is also visible in a distinctive peak in the distribution of adhesive states over the cell population. In Fig. 8, we show an example for a fixed *γ*-value of 0.25. The distribution in populations in which the adhesion phenotype is not under environmental control [ *α*=0, scenarios III and IV (CONTROL ^−^)] is almost normal and stays nearly constant over time [Fig. 8a and b]. In contrast, the distribution in populations of cells with density-dependent adhesion receptor regulation [ *α*=1, scenarios I and II (CONTROL ^+^)] is qualitatively differently for both *fast* and *slow regulation mode*: Whereas at the beginning of the simulation, the distribution also resembles the normal distribution [Fig. 8c, Additional file 10 (c)]; the distribution shifts towards a bimodal distribution with increasing simulation time [Fig. 8d, Additional file 10 (d)].

In summary, a higher intrinsic adhesion heterogeneity in a cell population shows two effects in our simulations. First, increasing adhesion heterogeneity leads to a higher number of disseminated cells. Second, the average of the adhesive states of disseminated cells decreases with increasing adhesion heterogeneity in the cell population. This causes higher adhesivity differences in the mean adhesion phenotypes between non-disseminated and disseminated cells.

### Sensitivity analysis for simulation parameters

We set the cell dissemination threshold distance to a fixed value of 50 in all simulations (Table 2), but also tested further values and found that the choice of the threshold distance is not important for the key results of our work (Additional file 11). Analogously, we did a sensitivity analysis for the number of rest channels per node *β* (Additional file 12) and the environmental control parameter *α* (Additional file 12). For all sensitivity analyses the heterogeneity parameter *γ*=0.25 remained fixed. We did not perform a sensitivity analysis for the constant adhesive state value in homogeneous populations because in our model cell-cell interaction only depends on the differences between the adhesive states of interacting cells (see Additional file 1 for details). As in homogeneous populations, all cells have constant and equal adhesive states, the absolute value does not change the model behaviour.

The three sensitivity analyses described above gave the following results: First, disseminated cells perform random walks and thus are more likely to be counted as disseminated cells if the threshold distance is short. This behaviour can be seen in lower r_diss_-values for higher threshold distances [Additional file 11 (a) and (b)] together with a decrease in mean adhesive states, indicating that cells with low equilibrium adhesive states have higher dissemination probabilities [Additional file 11 (c) and (d)]. Second, changing the number of channels *κ* | which affects the number of rest channels *β* | increases the number of stationary cells as the number of velocity channels stays constant. Accordingly, higher channel numbers are expected to generally reduce cell motility, which we found reflected in reduced r_diss_-values [Additional file 12 (a) and (b)]. Additionally, changing *κ* has no effect on the mean adhesive states [Additional file 12 (c) and (d)]. Third, the model behaves as expected for variations in the environmental control parameter *α* (Additional file 1). We show that for low *α*, the quantitative results converge towards independent adhesion regulation (corresponding to *α*=0) [Additional file 13 (c) and (d)]. For high *α*-values the quantitative results converge towards the density-dependent adhesion regulation (corresponding to *α*=1), reflecting the increasing sensitivity of adhesive states to changes in local cell density. For *γ*=0.25 changing *α* does not strongly affect the disseminated cell ratio r_diss_ [Additional file 13 (a) and (b)] as expected from Fig. 5 (*γ*=0.25).

Finally, we tested the effect of modelling intracellular adhesion receptor regulation in greater detail by comparing a *slow regulation mode*, in which we incorporate the adhesion receptor regulation model from Engwer et al. (2015) [49] with a *fast regulation mode*, in which we use a quasi-steady state approximation. For higher *γ*-values, the increase of r_diss_ is significantly lower for the *slow regulation mode* than for the *fast regulation mode* when compared for the same adhesion receptor regulation model (*γ*≥0.4, *p*<0.001). The finding that r_diss_ tends to be lower for populations with *slow regulation mode* in contrast to the *fast regulation mode* highlights the importance of also considering intracellular dynamics in modelling interacting cell populations [35]. Further support for this comes from our finding that the adhesion phenotype difference d_*a*_ is larger for the *slow regulation mode* than for the quasi-steady state approximation, even though this does not result in different distributions of mean adhesive states in the cell population (Additional file 10). These results also provide grounds for incorporating more realistic intracellular models into our multiscale model in the future, as in our model setting equilibrium states of the ODE are reached during simulations.

## Discussion

In this work, we propose a new multiscale model that combines a discrete LGCA model for adhesive cell-cell interaction and a continuous ODE model for intracellular regulation of adhesion receptor expression. The ODE model is used to calculate cellular adhesive states, which influence the adhesive interaction either independent from or depending on the local cell density.

In model simulations, we investigated the effect of adhesion heterogeneity on tumour cell dissemination from a circular initial cell configuration. The choice of this configuration is motivated by a densely packed epithelium and mimics in vitro invasion assays, as reported by Justus et al. [53]. To analyse our model, we determined the number of disseminated cells that migrate beyond a cell dissemination threshold distance within a given time and recorded the mean adhesive states of both non-disseminated and disseminated cells.

### Adhesion heterogeneity facilitates cell dissemination

We found that r_diss_, the ratio of disseminated cells at the end of a given time, significantly increases with the adhesion heterogeneity parameter *γ* (Fig. 5). Accordingly, adhesion heterogeneity in a cell population leads to an increase of the proportion of cells that disseminate from the population. Interestingly, we also found that r_diss_ is generally lower for populations, in which the adhesion receptor concentration increases with increasing local cell density, i.e. populations in which cellular adhesion phenotypes are under environmental control. This suggests that a positive correlation between adhesion receptor expression and local cell density increases cell-cell adhesion in epithelial cell populations, which weakens the effect of adhesion heterogeneity. Thus, environmental control of the adhesion phenotype could be a general mechanism to prevent cell dissemination from the cell population. Making this correlation negative so that adhesion receptor expression decreases with increasing local density lead to massive dispersal and dissemination of more than 90% of the cells in our model.

### Adhesion heterogeneity causes the emergence of two distinct subpopulations of cells

Adhesion heterogeneity does not only change the ratio r_diss_ of the number of disseminated cells to the total cell number, but also the mean adhesive states of disseminated cells. As expected, we found that non-disseminated cells have significantly higher mean adhesive states than disseminated cells. We used the difference between mean equilibrium adhesive states of non-disseminated and disseminated cells to define d_*a*_ as a distance measure between adhesion phenotypes. Our results show that d_*a*_ increases with an increasing adhesion heterogeneity. This is driven by lower mean adhesive states of disseminated cells $\bar {a}^{D}$ as the mean adhesive state of non-disseminated cells $\bar {a}^{N}$ is insensitive to the adhesion heterogeneity parameter *γ* (Fig. 7 and Additional file 3). This suggests that cells with low equilibrium $\bar {a}^{D}$-values, which have a higher probability to disseminate from the cell population, form a subpopulation of disseminated cells. Support for this interpretation comes from our observation in the sensitivity analysis of the cell dissemination threshold distance, which shows that higher threshold distances decrease equilibrium $\bar {a}^{D}$-values. In summary, for higher *γ*-values two subpopulations of cells emerge that can be distinguished by their respective mean adhesive states. These findings correlate well with the biological knowledge that EMT is accompanied by reduced E-cadherin expression [10, 14, 15] and that cells in metastases are often E-cadherin negative [18].

The distributions of adhesive states within the cell population exhibit a qualitative difference between the two adhesion receptor regulation models: Although the distribution follows a normal distribution for independent regulation, density-dependent regulation causes a bimodal distribution in which one peak can be associated with the initial cell population. The second peak at lower adhesive state values corresponds to disseminated cells (Fig. 8). This indicates that environmental control of the adhesion phenotype reinforces differences between cellular adhesion phenotypes. Thus, environmentally controlled adhesion phenotypes could lead to a trade-off between stronger adhesive coherence in a cell population and reduced adhesion receptor concentrations in disseminated cells. This offers an intriguing interpretation of our results: If cells with the lowest adhesion receptor concentration disseminate from the population and adhesion heterogeneity increases the number of these cells, adhesion heterogeneity could produce a constant flow of less adhesive disseminated cells with a rate that depends on the degree of heterogeneity. Accordingly, adhesion heterogeneity could be a mechanism that explains the occurrence of multiple disseminated cells within short periods of time or even during early tumour development. There is increasing evidence that tumour cells can disseminate in early, even pre-malignant stages, as opposed to the traditional concept that metastasis formation is a late event in tumour progression [54–56]. In line with this, our results suggest that adhesion heterogeneity can lead to tumour cell dissemination even in cells with sustained adhesion control.

### Adhesion heterogeneity could increase tumour cell dissemination efficiency

With our model, we study effects of adhesion heterogeneity on tumour cell dissemination. In doing so, we neglect several important factors, such as adhesive interaction of migrating cells with the extracellular matrix [10, 15], and other non-cellular environmental effects. A candidate for an environmental influence different from the studied cell density-dependent regulation is the impact of morphogen gradients, which are known to affect E-cadherin expression [57–59]. We also neglected cell division, since we were primarily interested in the isolated effects of adhesion heterogeneity on tumour cell dissemination. To relax the model assumption of non-dividing cells, however, would be of interest, given that the number of stem cell divisions strongly correlates with cancer risk over various tissues [60, 61], and that heterogeneity in cell division rates could also have great impact on tumour cell composition and therefore cell dissemination efficiency. We have previously shown that plasticity in proliferation/migration behaviour may have important consequences on tumour growth and invasion [44, 45, 48]. In the future, the mentioned factors, such as gradients and proliferation, can be included in our model one by one to determine their respective contribution to the complex phenomenon of tumour cell dissemination and ultimately tumour invasion, as LGCA models can be easily extended [50, 62–64]. Further, we decided to develop an LGCA on a 2D square lattice, which approximates the geometry of epithelial tissues well. In principle, the model definition can be extended to 1D or 3D lattices. Such extensions are potentially interesting for studying tumour cell dissemination in non-epithelial tissues, e.g. the 1D geometry is suitable for studying cell dissemination in breast ducts or small bronchi in the lungs and the 3D geometry is appropriate for studying corresponding phenomena in the brain.

Nevertheless, our proposed minimal model shows that adhesion heterogeneity reinforces tumour cell dissemination from dense cell populations of otherwise identical cells. Cell dissemination is reduced when adhesion receptor expression increases with local cell density, as can be assumed in healthy cells. When cells lose environmental control, i.e. adhesion receptor expression is independent from the environment, the rate of cell dissemination increases. This corresponds to tumour cells that gain independence from environmental signals. Interestingly, a scenario in which adhesion receptor expression decreases with increasing local density leads to massive dispersal of cells which could correspond to highly malignant tumour cells. In all cases, cell dissemination leads to the emergence of two distinct subpopulations, namely disseminated cells and cells that did not disseminate. These subpopulations can be distinguished by their mean adhesion receptor concentrations. The differences between the adhesion phenotypes are stronger when adhesion receptor expression of cells increases with increasing local cell density, i.e. if adhesion receptor expression is under environmental control. Following these simulation results, we hypothesise that adhesion heterogeneity, i.e. the differential expression of adhesion receptors between cells, provides a mechanism for more efficient metastasis. According to this hypothesis, we would expect that heterogeneity in adhesion receptor expression in cells of highly invasive tumours is larger than in less invasive types of cancer. Interestingly, recent experimental findings support our model prediction. In particular, disseminated tumour cells isolated from breast cancer patients show an extensive variability in the expression of epithelial cell adhesion molecules [65, 66].

Additionally, our simulation results suggest that adhesion receptor regulation in cells of tumours is independent from the local environment. To test this hypothesis, rigorous experimental investigation of E-cadherin expression levels in tumours and tumour metastases will be necessary. On the basis of such expression levels, one could distinguish effects of intrinsic and extrinsic regulation and parameterise the model.

Finally, as our model does not depend on assumptions with respect to cancer type or the type of surface molecules, our findings are not restricted to E-cadherin or epithelial cancers but could be used to describe a fairly general mechanism for any cell surface proteins that are involved in migration and invasion in various tumour types. For instance, expression levels of the epidermal growth factor receptor (EGFR) can be highly heterogeneous within glioblastoma multiforme, a particularly aggressive type of brain tumour [67]. EGFR is a cell surface protein that functions as an oncogene in many cancers due to aberrant activation and can promote invasion and migration in glioblastoma [68–70]. Even though targeting aberrations that are only altered in a subset of tumour cells can cause clonal selection and drug resistance, targeting EGFR variants can indeed prolong survival in glioblastoma patients, despite tumour recurrence [71]. Interestingly, the recurrent tumours tend to lack expression of the EGFR variant after therapy. Our model suggests a mechanism on the level of cell dissemination characteristics that explains how targeting EGFR aberrations, or heterogeneous aberrations, might increase treatment success based on the reduction of heterogeneity within the tumour. Our results suggest that strategies aimed at modulation migration are worth to be explored as alternatives to those mainly focused at keeping tumour proliferation under control.

## Conclusions

In this work, we analyse the effects of adhesion heterogeneity caused by intrinsic and extrinsic factors on the dissemination of tumour cells from an epithelial cell population theoretically. By studying a novel mathematical model, we report minimal conditions for the emergence of tumour invasion. In particular, we study the influence of adhesion heterogeneity and environmental control of adhesion receptor expression on tumour cell dissemination. In summary, our results indicate that reinforcement of cell dissemination in epithelial cell populations can be triggered by adhesion heterogeneity. If, however, adhesion receptor expression increases with the density of neighbouring cells, this effect can be compensated at least partially. Our findings offer an explanation for the promotion of tumour cell dissemination by both an increase in intra-tumour adhesion heterogeneity and hampered environmental control of adhesion receptor regulation. Based on these findings, we argue that adhesion heterogeneity provides a mechanism for more efficient metastasis that has already been partially confirmed by experimental studies.

## Reviewers’ comments

### Reviewer’s report 1: Hanspeter Herzel, ITB, Germany

**Reviewer comments:**

The introduction was easy to read and valuable references are provided. With the chapter LGCA model a stylistic break occurs. I am afraid that many non-experts stop reading here. The ODEs and the simulations later are better accessible to non-mathematicians than the operator concepts. I suggest to formulate the chapter less technical for a broad readership. Of course, precise technical details are required to ensure that the model is reproducible but technical aspects can be placed in the supplements.

Author’s response: *Following the reviewer’s suggestion, we have reformulated the model definition to make it accessible for a broad readership. We still provide the precise mathematical formalism in a supplementary note (Additional file* 1 *). With this additional information, readers are able to reproduce our findings.*

*Corresponding changes in the manuscript: p. 3-5 (defininition of the multiscale model and p. 4 (legend of Fig.* 1).

### Reviewer’s report 2: Thomas Dandekar, Department of Bioinformatics, University of Würzburg, Germany

**Reviewer comments: Major point 1**

First of all, cellular automata is an active field and it is reasonable to claim that for this particular question it is novel not just to use cellular automata (that has been demonstrated before, there are of course several models on cancer using this approach and the authors may re-check whether they want to give some other models in addition credit, no harm for the reader).

Author’s response: *We have added further examples of tumour-related studies using cellular automata and have reformulated the corresponding part in the introduction.*

*Corresponding changes in the manuscript: p. 2, 3.*

**Reviewer comments: Major point 2**

However, their lattice-gas cellular automaton model is elegantly implemented and in this sense at least for me novel: Exactly the right level of complexity to study basic properties of cell adhesion and if one looks closely at the involved equations the intricacies of the model become visible. 2b) Think however, that for instance you could also think about an alternative biology-inspired model such as “local growth – cell adhesion”. In such a model you assume that the local growth speed varies (not unreasonable, tumor cells can be less and more aggressive). If you then keep direct cell adhesion properties constant you will still have the thresholding behavior for larger cell masses as you currently have (nice feature, I liked that and good biology), as the more rapid dividing cells will easier reach the metastasis threshold. The interesting point is that you now have a completely different conclusion from your modeling: Only the growth speed determines whether you have metastasis or not and if you have there heterogeneity then this will lead to metastasis formation and the faster the more uneven the property of cell division is distributed in the original cancer population. Actually my impression is that this is indeed the case, that tumors which have very heterogenous cell division distribution such as lung cancer very rapidly develop metastasis and this is the main factor. Of course, due to this heterogeneity and, most of all, genetic instability of a highly malignant tumor (you may think for instance also on melanoma as another good example) you have then also heterogeneity of cell adhesion properties. However, in my mind, this is only a secondary indicator of the decisive principal factor, speed of cell division and that a cancer in which there is heterogeneity in this basic property is a really dangerous cancer, as all other sources of further heterogeneity such as cell adhesion derive from this. 2c– So maybe you can even check this alternative hypothesis in your model?

Author’s response: *We are aware that there are other factors that affect tumour dissemination and invasion, e.g. cancer cell proliferation, that are also worth studying. In fact, some of the authors have analysed effects of growth behaviour plasticity (in particular effects of the go or grow dichotomy) on tumour invasion in several studies (see e.g. ref. [40, 42] in our manuscript). The main goal of the current study was to focus on cell migration guided by adhesive interactions and to investigate the isolated effects of cellular adhesion heterogeneity within epithelial tumour cell populations on tumour cell dissemination that leads to tumour cell invasion and, ultimately, metastasis. According to the suggestions given by the reviewer, we discussed the motivation for our model choice in greater detail and point to the possibility to incorporate cell proliferation in future model extensions.*

*Corresponding changes in the manuscript: p. 12.*

**Reviewer comments: Major point 3**

The formalism is in a good sense theoretical: transparent mathematics, no unnecessary additional complexities and so one factor alone, in particular the cell adhesion, can be studied in transparent clarity. However, this is also the major limitation of the approach and my remaining comments pertain to the effort, to include even more biology in the article: For instance discuss a bit more, how now factors mentioned in the discussion such as EMT or gene expression data (you have their as a start figures mentioning extrinsic and intrinsic regulation) will be integrated in future versions of the model.

Author’s response: *Comprehensive experimental data on gene expression, especially adhesion receptor expression, heterogeneity is rare. We now refer to this lack of data more clearly in our manuscript, discuss some of the few available studies especially on EGFR regulation, and hint at the necessity of further studies (and how these might be designed) to experimentally support our findings. In particular, once available, expression data could be used to quantify effects of intrinsic and extrinsic regulation as predicted by our model.*

*Corresponding changes in the manuscript: p. 12, 13.*

**Reviewer comments: Major point 4**

What is nice in this model of the authors (and sufficient reason in my opinion to publish it and why I basically endorse it) is its elegance, but as given already in 1-3 the biology should still be further strengthened. For instance there are now two important papers by Bert Vogelstein which appeared in the last two years where he had this really impressive theory supported by data (!) that just the number of cell divisions alone in the stem cells enhances the probability to turn into cancer and that this fits well the observed different cancer probabilities for different cancer tissues. 4b–So the big question here is whether one can nail down the main factor in the model presented here, i.e. the assumed higher heterogeneity in cell adhesion properties for metastasis-bound tumors by some experimental data (for instance NGS data from tumor progression, take for instance the beautiful data from Francesca Cicarelli lab http://www.nature.com/articles/ncomms12072)? 4c–or maybe you rather find that my suggestion is right, heterogeneity in cell division? 4d–or that one pointed out by Prof. Cicarelli, i.e. damaging germline mutations in immune-related genes (where perhaps this latter feature is of course not fully integrated into your present model)? As you can see from these comments, there is nothing wrong with your nice modeling approach, but on the contrary, rather I consider it strong enough to probe some of the more challenging questions in tumorigenesis and metastasis and of course develop the approach further.

Author’s response: *The main prediction of our modelling study is that adhesion heterogeneity can promote cancer cell dissemination and invasion and therefore is an indicator of malignancy. Thanks to the reviewer’s suggestion, we have now included a reference to brand new studies on breast cancer which support the model prediction of a positive correlation between molecular heterogeneity and malignancy.*

*Corresponding changes in the manuscript: p. 12.*

### Reviewer’s report 3: Marek Kimmel, Rice University, USA

**Reviewer comments: Major point 1**

It will help the paper if the authors explain several fundamental issues, which mostly concern the realism of this model. 1. The model does not include proliferation, which is an important element of seeding of metastases, although the authors discuss the role of EGFR in cancer progression.

Author’s response: *We are aware that there are other factors that affect tumour dissemination and invasion, e.g. cancer cell proliferation, that are also worth studying. In fact, some of the authors have analysed effects of growth behaviour plasticity (in particular effects of the go or grow dichotomy) on tumour invasion in several studies (see e.g ref. [40, 42] in our manuscript). The main goal of the current study was to investigate the isolated effect of cellular adhesion heterogeneity within epithelial tumour cell populations on tumour cell dissemination that leads to tumour cell invasion and, ultimately, metastasis. According to the suggestions given by the reviewer, we discussed the reasons for our model choice in greater detail and point to the possibility to incorporate cell proliferation in a future model extension. (see also our response to Thomas Dandekar, major point 2).*

*Moreover, we have more clearly formulated the postulated role of EGFR in our model.*

*Corresponding changes in the manuscript: p. 12.*

**Reviewer comments: Major point 2**

On the technical side, the automaton includes up to 10 cells in each node, each of them in one of the four active or one of the six resting channels. It is not quite clear what is the role of such configuration. If I understand correctly, in the basic gas lattice automaton the state of the node is simply binary. 3. Again, as far as I understand the model, this is a planary (two-dimensional) automaton. If this is correct, it is a far idealization of the three-dimensional reality. On the other hand, cancer spread frequently occurs along tubular or linear structures such as breast ducts or small bronchi in the lungs. Accordingly, it might help if the authors discuss the role of geometry.

Author’s response: *Following the reviewer’s suggestion, we described the node configuration in greater detail and also motivated our choice of a 2D geometry.*

*Corresponding changes in the manuscript: p. 12/13.*

## Additional files

Additional file 1 Model details. Mathematical formalism and full model description. (PDF 225 kb)

Additional file 2LGCA transition dynamics. LGCA transition dynamics $\mathcal {D}:=\mathcal {R} \, \circ \, \mathcal {A}$ in a *von Neumann* neighbourhood $\mathcal {N}_{\boldsymbol {r}}$ around node ***r*** (gray) followed by translocation $\mathcal {T}_{i}$. (a) During reorientation $\mathcal {R}$ cells are stochastically redistributed within nodes according to a probability function *P*. The highest probability is assigned to a resulting node configuration ***η*** ^′^(***r***)with post-reorientation momentum ***J***:=***J***(***η*** ^′^,***a*** ^″^)(***r***)parallel to the pre-reorientation local adhesivity gradient ***G***:=***G***(***η***,***a*** ^′^)(***r***)of the neighbourhood excluding ***r*** [Fig. 1b]. (b) In our model, the newly introduced adhesivity update operator $\mathcal {A}$ couples the time scales of the LGCA and ODE models. The graph shows an example for the analytical solution *y* ^***r***^(*t*)[Eq. (1)] of the underlying ODE [Eq. 1] according to which $\mathcal {A}$decreases the adhesive states *a*_*i*_(***r***,*k*)of all cells as *a*_*i*_(***r***,*k*)>*a*_*i*_(***r***,*k*+*τ*) ∀ *k*; in this example there is no adhesion heterogeneity. The update from time-step *k* = *t* to *k*+*τ* = *t*+*τ* shown here is labelled red in the graph. After reorientation, cells are moved by the translocation operator $\mathcal {T}_{i}$ (see Additional file 1 for details). Note that all nodes have only one rest channel. Notation as in Fig. 1. (PDF 28 kb)

Additional file 3Mean adhesive states comparison. Mean adhesive states comparison for disseminated cells in populations with different adhesion heterogeneity parameters ***γ*** and *α*=1. The plots show the mean adhesive states [Eq. (4)] of disseminated cells $\bar {a}^{D}$ (curves) and non-disseminated cells $\bar {a}^{N}$ (curves + circles) over time for *γ*=0,0.05,0.25,0.4,0.55 and *α*=1. Both $\bar {a}^{D}$ and $\bar {a}^{N}$ converge towards equilibrium states for all *γ*-values. The decrease of mean equilibrium adhesive states with increasing *γ*-values is significantly stronger between and for fixed *γ* in scenarios in which cellular adhesion phenotypes are under environmental control (red, Additional files 5 and 6). (PNG 347 kb)

Additional file 4 Full statistics for Fig. 5b (1). *p*-values from two-tailed t-tests for significance levels in Fig. 5b between *γ*-values. *p* ^*w*^-values were calculated with Welch tests as variances between samples were significantly (*p* ^*f*^<0.05) different. (XLSX 37 kb)

Additional file 5 Full statistics for Fig. 5b (2). *p*-values from two-tailed t-tests for significance levels in Fig. 5b for fixed *γ*-values. *p* ^*w*^-values were calculated with Welch tests as variances between samples were significantly (*p* ^*f*^<0.05) different. (XLSX 32 kb)

Additional file 6 Full statistics for Fig. 7a (1). *p*-values from two-tailed t-tests for significance levels in Fig. 7a between *γ*-values. *p* ^*w*^-values were calculated with Welch tests as variances between samples were significantly (*p* ^*f*^<0.05) different. (XLSX 37 kb)

Additional file 7 Full statistics for Fig. 7a (2). *p*-values from two-tailed t-tests for significance levels in Fig. 7a for fixed *γ*-values. *p* ^*w*^-values were calculated with Welch tests as variances between samples were significantly (*p* ^*f*^<0.05) different. (XLSX 32 kb)

Additional file 8 Full statistics for Fig. 7b (1). *p*-values from two-tailed t-tests for significance levels in Fig. 7b between *γ*-values. *p* ^*w*^-values were calculated with Welch tests as variances between samples were significantly (*p* ^*f*^<0.05) different. (XLSX 37 kb)

Additional file 9 Full statistics for Fig. 7b (2). *p*-values from two-tailed t-tests for significance levels in Fig. 7b for fixed *γ*-values. *p* ^*w*^-values were calculated with Welch tests as variances between samples were significantly (*p* ^*f*^<0.05) different. (XLSX 32 kb)

Additional file 10Equilibrium mean adhesive state distributions. Equilibrium mean adhesive state distributions in cell populations with adhesion heterogeneity (*γ*=0.25) and *slow regulation mode*. (a) and (b) show the distributions of mean adhesive states $\bar {a}$ in equilibrium for cell populations with only intrinsic adhesion heterogeneity and the *slow regulation mode* at time *k*=1 and *k*=1000 (Scenario I). (c) and (d) show the distributions of $\bar {a}$ in equilibrium for cell populations with additional extrinsic adhesion heterogeneity and the *slow regulation mode* at time *k*=1 and *k*=1000 (Scenario III). The equilibrium adhesivity distributions do not differ when the *fast regulation mode* is considered (Fig. 8). (PNG 7966 kb)

Additional file 11Sensitivity to the cell dissemination threshold distanc. Sensitivity to the cell dissemination threshold distance for *γ*=0.25. (a) shows the disseminated cell ratio r_diss_ over time for different threshold distances. (b) shows r_diss_ over time for different threshold distances in a higher resolution for low values. From both (a) and (b) one can see that r_diss_ decreases with the cell dissemination threshold distance as would be expected. There is a striking difference for the cell dissemination threshold distance of 5 where r_diss_ increases drastically until it saturates at high ratios. (c) shows the mean equilibrium adhesive state of disseminated cells $\bar {a}^{D}$ for different threshold distances. (d) shows the difference in adhesion phenotypes d_*a*_ between the two subpopulations for different threshold distances. As would be expected, the adhesion phenotype does not strongly depend on the distance threshold except for a very low distance threshold of 5. In the latter case more than half of the cells are considered disseminated so that the differentiation between the adhesion phenotypes is blurred. This is not surprising as within such short distance cells are likely to disseminate and re-join the cell population due to stochasticity. Accordingly, the effect is rather a model artefact than a biological phenomenon. (PNG 8294 kb)

Additional file 12Sensitivity to the number of channel. Sensitivity to the number of channels ***κ*** for *γ*=0.25. (a) shows the maximum value of the disseminated cell ratio r_diss_ for different values of *κ*that affect the number of rest channels *β*. (b) shows r_diss_ over time for different values of *κ*. Both the maximum of r_diss_ and the slope of r_diss_ as a function of time decrease with *κ* as expected. This is due to lower mobility caused by a lower numbers of rest channels. (c) shows the mean equilibrium adhesive state of disseminated cells $\bar {a}^{D}$ for different values of *κ*. (d) shows the difference in adhesion phenotypes d_*a*_ between the two subpopulations for different values of *κ*. As expected, the adhesion phenotype does not depend on *κ*. (PNG 8058 kb)

Additional file 13Sensitivity to the environmental control parameter. Sensitivity to the environmental control parameter *α* for *γ*=0.25. (a) shows the maximum value of the disseminated cell ratio r_diss_ for different values of *α*. (b) shows r_diss_ over time for different values of *α*. Both the maximum of r_diss_ and r_diss_ over time do not depend on *α*. (c) shows the mean equilibrium adhesive state of disseminated cells $\bar {a}^{D}$ for different values of *α*. (d) shows the difference in adhesion phenotypes d_*a*_ between the two subpopulations for different values of *α*. Whereas the mean equilibrium value of $\bar {a}^{D}$ decreases with *α*, the distance d_*a*_ between the adhesion phenotypes increasesdue to growing influence of the environmental control mechanism (Fig. 7). (PNG 7925 kb)

## Abbreviations

ECM: Extracellular matrix
EGFR: Epidermal growth factor receptor
EMT: Epithelial-mesenchymal transition
LGCA: Lattice-gas cellular automaton
ODE: Ordinary differential equation
2D: 2-dimensional
