# Supplementary material for: Cell adhesion heterogeneity reinforces tumour cell dissemination: novel insights from a mathematical model
Source: Biol Direct. 2017 Aug 11;12:18. doi: 10.1186/s13062-017-0188-z (PMC5553611; doi:10.1186/s13062-017-0188-z)
Supplement: Supplementary file 1 — Model details. Mathematical formalism and full model description. (PDF 225 kb) [file 13062_2017_188_MOESM1_ESM.pdf]

RESEARCH

Additional File 1: Cell adhesion heterogeneity reinforces tumour cell dissemination: novel insights from a mathematical model - Model details

David Reher[1,2*], Barbara Klink[3,4], Andreas Deutsch[2] and Anja Voss-Böhme[2,5]

*Correspondence: david_reher@eva.mpg.de
[1]Department of Evolutionary Genetics, Max Planck Institute for Evolutionary Anthropology, Deutscher Platz 6, 04103 Leipzig, Germany
[2]Center for Information Services and High Performance Computing, Technische Universität Dresden, Nöthnitzer Str. 46, 01062 Dresden, Germany
Full list of author information is available at the end of the article

Formal description of the LGCA model

LGCA model

The LGCA model is defined on a discrete 2-dimensional square lattice $\mathcal{L}$ with periodic boundary conditions [2, 3]. The lattice-gas model used in our work is an extension of cellular automata with binary states that has first been used in statistical physics and fluid mechanics (see [1] for an overwiew). Each lattice node $\boldsymbol{r} \in \mathcal{L}$ is connected to its four nearest neighbours, forming its *von Neumann* neighbourhood $\mathcal{N}_{\boldsymbol{r}}$, by unit vectors $\boldsymbol{c}_i, i = 0, ..., 3$, called velocity channels. The total number of channels per node is defined by κ, ands $\beta := \kappa - 4$ is an arbitrary number of channels with zero velocity, called rest channels, in which $\boldsymbol{c}_i = 0$, $4 \leq i < \kappa$. Each channel can be occupied by at most one cell at a time. In occupied channels, the occupation state $\eta_i(\boldsymbol{r}) = 1, i = 1, ..., \kappa$, whereas for empty channels $\eta_i(\boldsymbol{r}) = 0$. If $\eta_i(\boldsymbol{r}) = 1$, the occupying cell's adhesive state is described by the variable $a_i(\boldsymbol{r}) \in \mathbb{R}^+ := [0, \infty)$. Occupation states $\eta_i(\boldsymbol{r})$ and adhesive states $a_i(\boldsymbol{r})$ of all channels in a node $\boldsymbol{r}$ give the node configuration $(\boldsymbol{\eta}, \boldsymbol{a})(\boldsymbol{r})$, formally defined as $(\boldsymbol{\eta}, \boldsymbol{a})(\boldsymbol{r}) := ((\eta_0, ..., \eta_{\kappa-1}), (a_0, ..., a_{\kappa-1}))(\boldsymbol{r}) \in \mathcal{E}_a := \{0, 1\}^\kappa \times {\mathbb{R}^+}^\kappa$. Fig. 1 (a) illustrates the state space of the LGCA model.

LGCA dynamics are characterised by a transition operator $\mathcal{D} : \mathcal{E}_a \to \mathcal{E}_a, (\boldsymbol{\eta}, \boldsymbol{a})(\boldsymbol{r}) \mapsto (\boldsymbol{\eta}', \boldsymbol{a}'')(\boldsymbol{r})$, that updates a given node configuration $(\boldsymbol{\eta}, \boldsymbol{a})(\boldsymbol{r}, k) := (\boldsymbol{\eta}, \boldsymbol{a})(\boldsymbol{r})$ to a subsequent node configuration $(\boldsymbol{\eta}, \boldsymbol{a})(\boldsymbol{r}, k+\tau) := (\boldsymbol{\eta}', \boldsymbol{a}'')(\boldsymbol{r})$ at time $k+\tau \in \mathcal{K}$ and is simultaneously applied to each node $\boldsymbol{r} \in \mathcal{L}$ at discrete time $k \in \mathcal{K} := \{j\,\tau \mid j \in \mathbb{N}\}$. The time-step length $\tau \in \mathbb{R}^+$, $\tau > 0$ is constant.

We define $\mathcal{D}$ as the composition of two operators:

- The **deterministic adhesivity change operator**

$$\mathcal{A} : (\boldsymbol{\eta}, \boldsymbol{a})(\boldsymbol{r}) \to (\boldsymbol{\eta}, \boldsymbol{a}')(\boldsymbol{r}) \tag{1}$$

  calculates new adhesive states $a'_i(\boldsymbol{r})$ for every cell at node $\boldsymbol{r}, 0 \leq i < \kappa$. To determine the new values for the adhesive states, we use an intracellular adhesion receptor regulation model on the basis of an ordinary differential equation (ODE) described below [eq. (4)].

- The **probabilistic reorientation operator**

$$\mathcal{R} : (\boldsymbol{\eta}, \boldsymbol{a}')(\boldsymbol{r}) \to (\boldsymbol{\eta}', \boldsymbol{a}'')(\boldsymbol{r}) \tag{2}$$

  redistributes cells together with their adhesive states within a node $\boldsymbol{r}$ according to a probability function P described below [eq. (14)].

Accordingly, $\mathcal{D} := \mathcal{R} \circ \mathcal{A}$.
After reorientation, cells in velocity channel $\boldsymbol{c}_i$ of node $\boldsymbol{r}$ are deterministically moved to channel $\boldsymbol{c}_i$ of the neighbouring node $\boldsymbol{r} + \boldsymbol{c}_i \in \mathcal{L}$ according to the translocation operator $\mathcal{T}_i : (\boldsymbol{\eta}', \boldsymbol{a}'')(\boldsymbol{r}) \mapsto (\boldsymbol{\eta}', \boldsymbol{a}'')(\boldsymbol{r} + \boldsymbol{c}_i)$ (see Additional file 7 for details) that is defined by

$$\mathcal{T}_i : (\eta_i', a_i'')(\boldsymbol{r}) := (\eta_i', a_i'')(\boldsymbol{r} + \boldsymbol{c}_i), \qquad i = 0, ..., \kappa - 1, \boldsymbol{r} \in \mathcal{L}. \tag{3}$$

Deterministic intracellular adhesion receptor regulation model
We describe the adhesion receptor concentration of individual cells positioned at $(\boldsymbol{r}, \boldsymbol{c}_i)$ at time k by an adhesive state variable $a_i(\boldsymbol{r}, k)$. To determine $a_i(\boldsymbol{r}, k)$, we use the following ODE [adapted from [4]]:

$$\frac{dy_i^{\boldsymbol{r}}(t)}{dt} = h^+(R_0 - y_i^{\boldsymbol{r}}(t)) - h^- y_i^{\boldsymbol{r}}(t) \tag{4}$$

with $y_i^{\boldsymbol{r}}(t)$ the concentration of adhesion receptors on the cell surface at continuous time $t \in \mathbb{R}_0^+$, $h^+, h^- \in \mathbb{R}$ the respective adhesion receptor association and dissociation rates, $R_0 \in \mathbb{N}$ the maximum adhesion receptor concentration. The initial condition is $y_i^{\boldsymbol{r}}(0) = y_0$ (see Table 1 in main text for chosen parameter values).
The solution of eq. (4) can be obtained analytically and is given by

$$y_i^{\boldsymbol{r}}(t) = c\,\mathrm{e}^{-(h^+ + h^-)t} + \frac{h^+ R_0}{h^+ + h^-}, \tag{5}$$

where $c \in \mathbb{R}$ is a constant of integration. Setting $t = 0$ gives

$$c = y_0 - \frac{h^+ R_0}{h^+ + h^-}, \tag{6}$$

where y_0 is the initial adhesion receptor concentration. The steady state of the ODE model [eq. 4] is given by $\frac{h^+ R_0}{h^+ + h^-}$.
We distinguish between *fast* and *slow* intracellular adhesion receptor regulation. For the *fast regulation mode*, we use a quasi-steady state approximation and assume that the steady state is reached almost instantly. In this case, we $y_i^{\boldsymbol{r}}(t)$ to $\frac{h^+ R_0}{h^+ + h^-}$ for $t \geq 0$.
For the *slow regulation mode*, we calculate an adhesive state according to the analytical solution of the ODE model [eq. (5)] for every discrete cellular automaton

time k and every cell. The continuous adhesion receptor concentration $y_i^{\boldsymbol{r}}(t)$ of a cell at $(\boldsymbol{r}, \boldsymbol{c}_i)$ is temporally discretised to give the adhesive state variable $a_i(\boldsymbol{r}, k)$ by passing the discrete time-step of the LGCA model to eq. (5) as an argument [Fig. 2 (a) and Additional file 7 (b)]. For the temporal update, let $a_i(\boldsymbol{r}, k+\tau)$ be the adhesive state at time $k + \tau \in \mathcal{K}$.

Heterogeneity in the intracellular adhesion receptor regulation model
We introduce intrinsic adhesion heterogeneity by assigning independent stochastic values to two ODE parameters, the initial adhesive state y_0 and the maximum adhesive state R_0 (Fig. 2). Heterogeneity in these parameters is achieved by randomly drawing values from a normal distribution for each cell before starting the simulation. The respective expected values $\langle y_0 \rangle$ and $\langle R_0 \rangle$ are fixed (Tab. 1 in main text). As a control parameter for the degree of heterogeneity, we use the coefficient of variation and denote it by γ

$$\gamma = \frac{\sigma_{y_0}}{< y_0 >} = \frac{\sigma_{R_0}}{< R_0 >} \tag{7}$$

where σ_{y_0} and σ_{R_0} are the standard deviations of y_0 and R_0, respectively. γ-values are chosen to be equal for y_0 and R_0. The rates h^+ and h^- are held constant and identical for all cells. Note that rates h^+ and h^- have different units compared to rates of second order reactions as $y_i^{\boldsymbol{r}}(t)$ is not a molar concentration but the actual number of adhesion receptors on the cell surface [4]. For the *fast regulation mode*, where we approximate eq. (4) by the steady state value, the parameter R_0 that determines the steady state value $\frac{h^+ R_0}{h^+ + h^-}$ is drawn from a normal distribution with the same parameters as above.
For modelling extrinsic cell density-dependent adhesion receptor regulation, we modify eq. (5) by considering a linear cell density-dependent weight. To account for changes in cell density within the circular core population, we normalise the local cell density with the average global cell density, such that

$$y_i^{\boldsymbol{r}}(t, \rho(\mathcal{N}_{\boldsymbol{r}}, k)) = \left[1 - \alpha + \alpha \left(\frac{\rho(\mathcal{N}_{\boldsymbol{r}}, k)}{\bar{\rho}(N, k)}\right)\right] \; y_i^{\boldsymbol{r}}(t), \quad \alpha \in [0, 1], \tag{8}$$

where α is an environmental control parameter and $\bar{\rho}(N, k)$ is the global average cell population density, defined as

$$\bar{\rho}(N, k) := \frac{1}{N} \sum_{\boldsymbol{r}=1}^{N} \sum_{i=0}^{\kappa-1} \frac{1}{\kappa} \, \eta_i(\boldsymbol{r}, k) \tag{9}$$

with $N := N(\boldsymbol{r}, k)$ the number of nodes in $\mathcal{L}$ with at least one occupied channel at time $k \in \mathcal{K}$ and, as before, κ the number of channels per node. The term

$$\rho(\mathcal{N}_{\boldsymbol{r}}, k) := \frac{1}{5} \sum_{j=0}^{4} \sum_{i=0}^{\kappa-1} \frac{1}{\kappa} \, \eta_i(\boldsymbol{r} + \boldsymbol{c}_j, k) \qquad \in \mathbb{R} \tag{10}$$

describes the local cell density in a neighbourhood $\mathcal{N}_{\boldsymbol{r}}$ at time $k \in \mathcal{K}$. To model a decrease in adhesive states with increasing cell density, we changed eq. (8) such that the density-dependent weighting term linearly decreases with increasing local cell density, i.e.

$$y_i^{\boldsymbol{r}}(t, \rho(\mathcal{N}_{\boldsymbol{r}}, k)) = \left[1 - \alpha \left(\frac{\rho(\mathcal{N}_{\boldsymbol{r}}, k)}{\bar{\rho}(N, k)}\right) + \alpha\right] \; y_i^{\boldsymbol{r}}(t), \quad \alpha = 1. \tag{11}$$

Probabilistic migration step guided by intracellular adhesion receptor concentration
To account for adhesive interaction between cells, we model a probabilistic preference of migration towards areas with high local cell densities, i.e. nodes with high cell numbers $n_{\boldsymbol{\eta}(\boldsymbol{r})} := \sum_{i=0}^{\kappa-1} \eta_i(\boldsymbol{r})$. Thereby, the strength of adhesive interactions depends on the adhesive states $a_i(\boldsymbol{r}, k)$ of the interacting cells. We weight the cell numbers by the adhesive states $a_i(\boldsymbol{r}, k)$ of the interacting cells. This gives a momentum $\boldsymbol{J} := \boldsymbol{J}(\boldsymbol{\eta}, \boldsymbol{a})(\boldsymbol{r})$ of a node configuration $(\boldsymbol{\eta}, \boldsymbol{a})(\boldsymbol{r})$, defined by

$$\boldsymbol{J}(\boldsymbol{\eta}, \boldsymbol{a})(\boldsymbol{r}) := \sum_{i=0}^{\kappa-1} \boldsymbol{c}_i \, \eta_i(\boldsymbol{r}) \, a_i(\boldsymbol{r}). \tag{12}$$

The vector sum of all momenta in $\mathcal{N}_{\boldsymbol{r}} \setminus \{\boldsymbol{r}\}$ gives a local adhesivity gradient $\boldsymbol{G}(\boldsymbol{\eta}, \boldsymbol{a})(\boldsymbol{r})$ around node $\boldsymbol{r} \in \mathcal{L}$, excluding $\boldsymbol{r}$ (Fig. 1), defined by

$$\boldsymbol{G}(\boldsymbol{\eta}, \boldsymbol{a})(\boldsymbol{r}) := \sum_{j=0}^{3} \sum_{i=0}^{\kappa-1} \boldsymbol{c}_j \, \eta_i(\boldsymbol{r} + \boldsymbol{c}_j) \, a_i(\boldsymbol{r} + \boldsymbol{c}_j). \tag{13}$$

The reorientation probability $P : (\boldsymbol{\eta}, \boldsymbol{a}')(\boldsymbol{r}) \to (\boldsymbol{\eta}', \boldsymbol{a}'')(\boldsymbol{r})$ depends on the post-reorientation momentum $\boldsymbol{J} := \boldsymbol{J}(\boldsymbol{\eta}', \boldsymbol{a}'')(\boldsymbol{r})$ and the pre-reorientation local adhesivity gradient $\boldsymbol{G} := \boldsymbol{G}(\boldsymbol{\eta}, \boldsymbol{a}')(\boldsymbol{r})$. To model adhesive interaction as attraction between cells depending on their adhesive states, we define the reorientation probability P such that it increases with the degree of alignment between $\boldsymbol{J}$ and $\boldsymbol{G}$ [Fig. 1 (b)]. Formally, we achieve this by using the scalar product of $\boldsymbol{J}$ and $\boldsymbol{G}$. We then define the reorientation probability P such that, at each node $\boldsymbol{r} \in \mathcal{L}$,

$$P((\boldsymbol{\eta}, \boldsymbol{a'}(\boldsymbol{r})) \to (\boldsymbol{\eta}', \boldsymbol{a}'')(\boldsymbol{r}))) := \frac{1}{Z(\boldsymbol{\eta}, \boldsymbol{a'})} \; \exp(\langle \boldsymbol{J}, \boldsymbol{G} \rangle) \, \delta_{\boldsymbol{\eta}\,\boldsymbol{\eta}'} \, \Pi_{\boldsymbol{a}'\,\boldsymbol{a}''}. \tag{14}$$

With Kronecker's delta $\delta_{\boldsymbol{\eta}\,\boldsymbol{\eta}'}$ defined as

$$\delta_{\boldsymbol{\eta}\,\boldsymbol{\eta}'} := \delta(n_{\boldsymbol{\eta}}, n_{\boldsymbol{\eta}'}) = \begin{cases} 1 & : \quad n_{\boldsymbol{\eta}} = n_{\boldsymbol{\eta}'} \\ 0 & : \quad \text{else}, \end{cases} \tag{15}$$

we ensure that the number of cells at each node $\boldsymbol{r}$ before reorientation $n_{\boldsymbol{\eta}}$ is equal to the number of cells after reorientation $n_{\boldsymbol{\eta}'}$, i.e the number of cells in $\boldsymbol{r}$ stays

constant during reorientation.
The function $\Pi_{\boldsymbol{a}'\,\boldsymbol{a}''}$ ensures that the adhesive states of all rearranged cells within the channels of a given node $\boldsymbol{r}$ are maintained. It is defined as

$$\Pi_{\boldsymbol{a}'\,\boldsymbol{a}''} := \begin{cases} 1 & : \quad a'_{\pi(i)} = a_i,\ i = 0, ..., \kappa - 1 \text{ for a permutation } \pi \text{ of } (0, ..., \kappa - 1) \\ 0 & : \quad \text{else.} \end{cases} \tag{16}$$

The term $Z(\boldsymbol{\eta}, \boldsymbol{a'})$ is a normalisation term such that P is indeed a probability. It is given by

$$Z(\boldsymbol{\eta}, \boldsymbol{a}) := \sum_{\boldsymbol{\eta}' \in \mathcal{E}_a} \exp(\langle \boldsymbol{J}, \boldsymbol{G} \rangle)\, \delta_{\boldsymbol{\eta}\,\boldsymbol{\eta}'}\, \Pi_{\boldsymbol{a}'\,\boldsymbol{a}''}. \tag{17}$$

Author details
[1]Department of Evolutionary Genetics, Max Planck Institute for Evolutionary Anthropology, Deutscher Platz 6, 04103 Leipzig, Germany. [2]Center for Information Services and High Performance Computing, Technische Universität Dresden, Nöthnitzer Str. 46, 01062 Dresden, Germany. [3]Institute for Clinical Genetics, Faculty of Medicine Carl Gustav Carus, Technische Universität Dresden, Fetscherstr. 74, 01307 Dresden, Germany. [4]German Cancer Consortium (DKTK), Dresden, Germany; German Cancer Research Center (DKFZ), Heidelberg, Germany; Center for Molecular Tumor Diagnostics (CMTD), National Center for Tumor Diseases (NCT), Dresden, Germany. [5]Hochschule für Technik und Wirtschaft Dresden, Fakultät Informatik/Mathematik, Friedrich-List-Platz 1, 01069 Dresden, Germany.

References

1. Deutsch, A., Dormann, S.: Cellular Automaton Modeling of Biological Pattern Formation. Birkhäuser, Basel (2005, 2nd ed. 2017)
2. Mente, C.: Tracking of individual cell trajectories in LGCA models of migrating cell populations. PhD thesis, TU Dresden (2015)
3. Mente, C., Voss-Böhme, A., Deutsch, A.: Analysis of individual cell trajectories in lattice-gas cellular automaton models for migrating cell populations. B Math Biol **77**(4), 660–697 (2015)
4. Engwer, C., Hillen, T., Knappitsch, M., Surulescu, C.: Glioma follow white matter tracts: a multiscale DTI-based model. J Math Biol **71**(3), 551–582 (2015)
