# Supplementary figures and images for: Cell adhesion heterogeneity reinforces tumour cell dissemination: novel insights from a mathematical model
Source: Biol Direct. 2017 Aug 11;12:18. doi: 10.1186/s13062-017-0188-z (PMC5553611; doi:10.1186/s13062-017-0188-z)

(a)

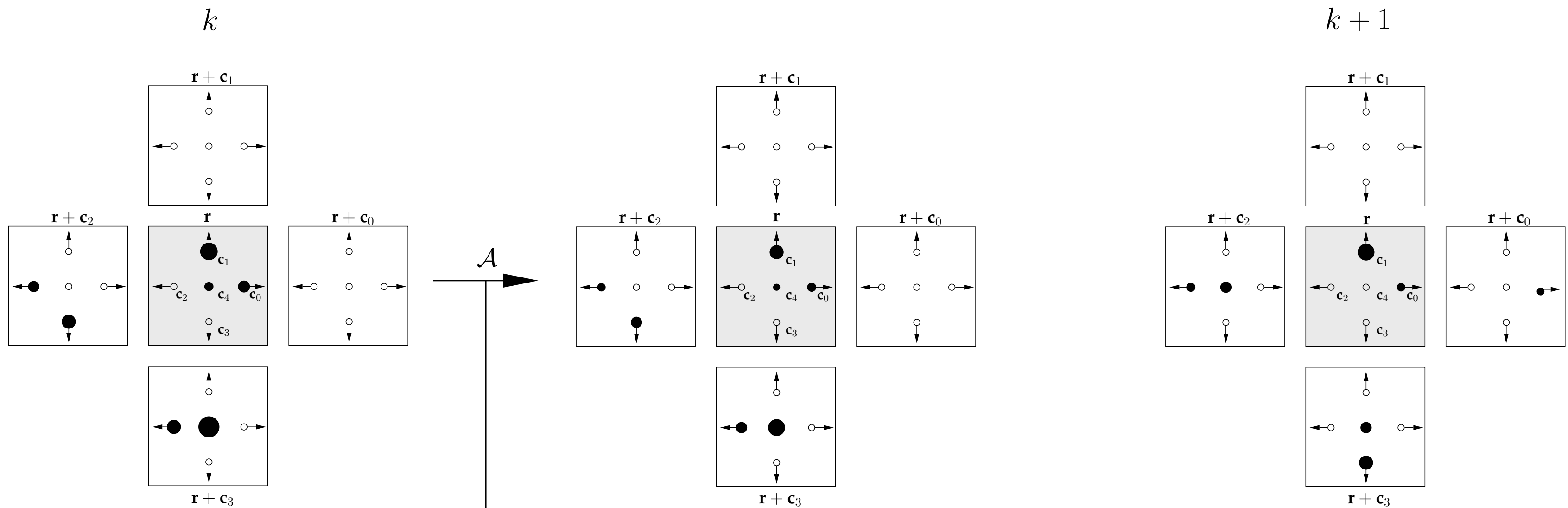

(b)

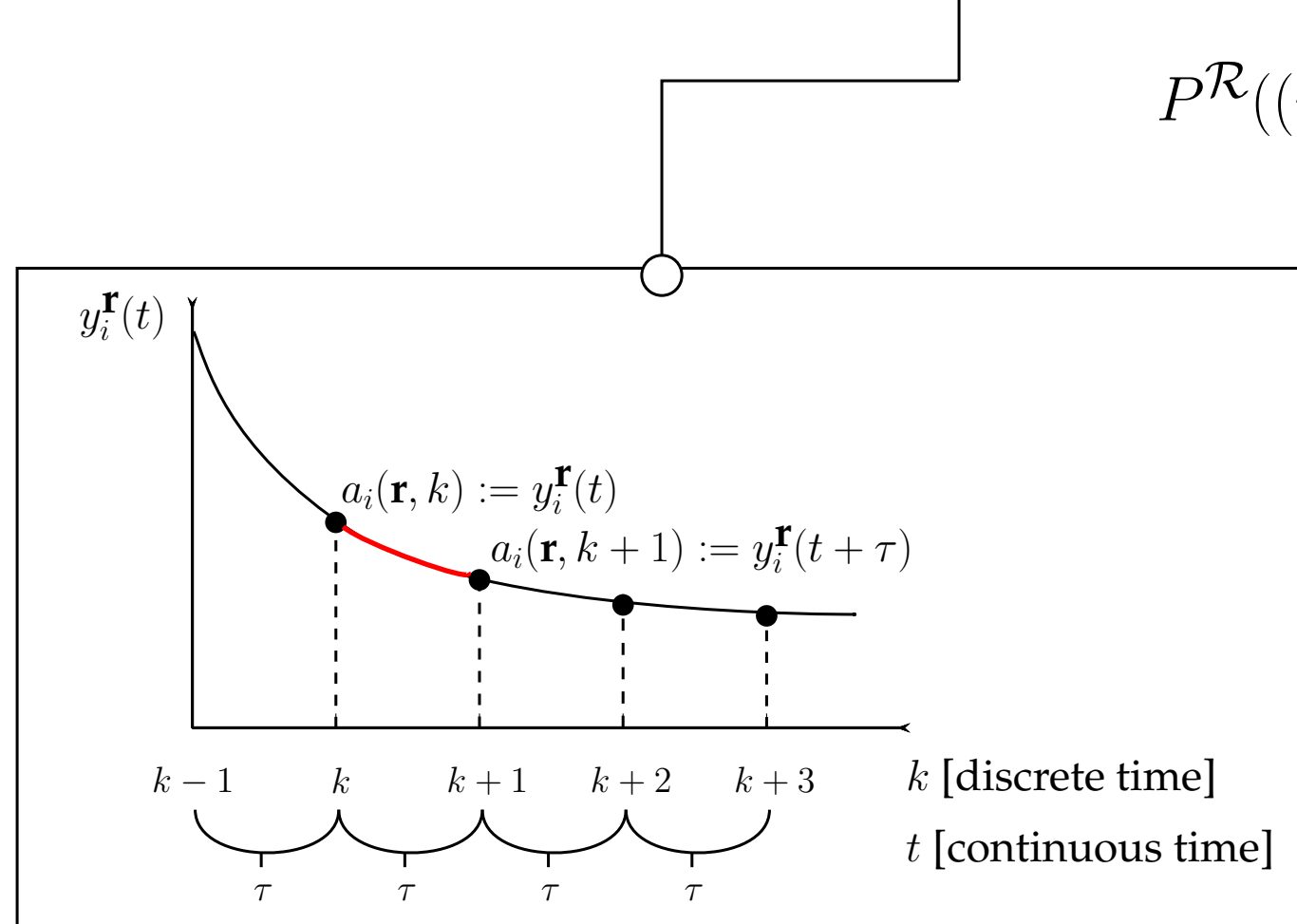

$$P^{\mathcal{R}}((\eta_{\mathcal{N}_r^4}, a_{\mathcal{N}_r^4})(r))$$

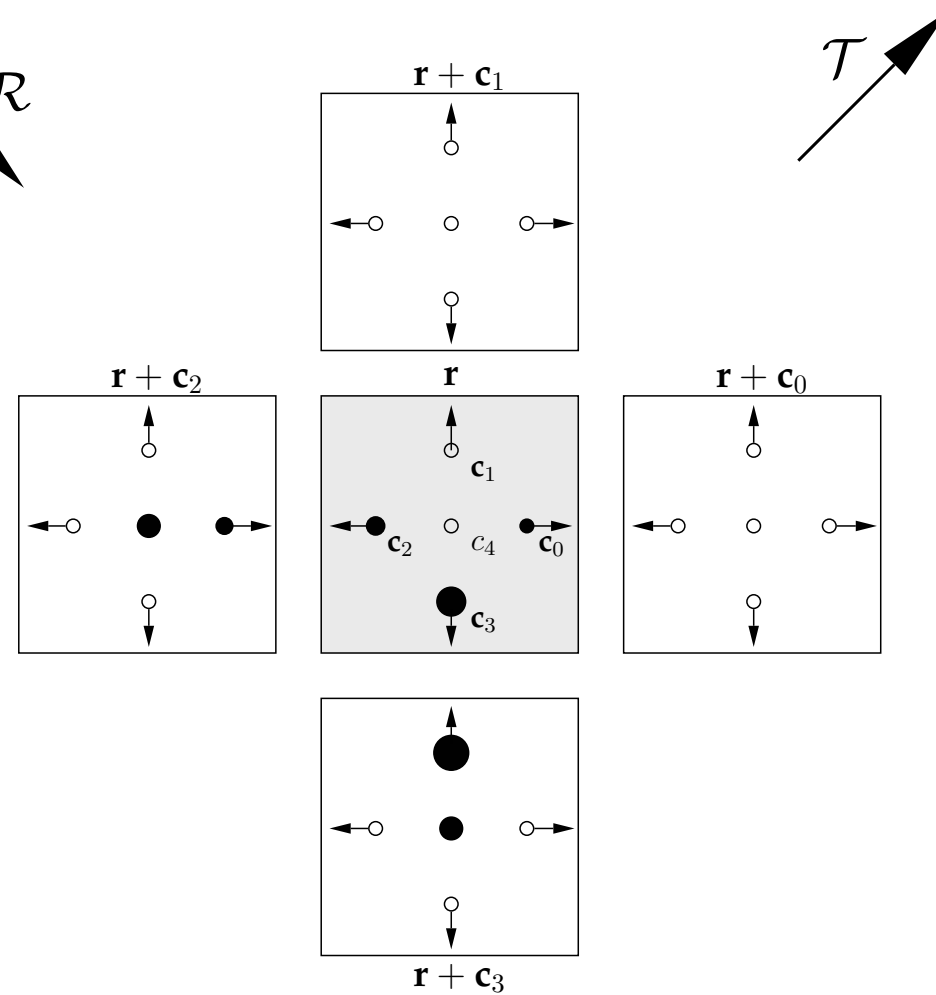

Supplement: Supplementary file 2 — LGCA transition dynamics. LGCA transition dynamics $\mathcal {D}:=\mathcal {R} \, \circ \, \mathcal {A}$D:=R∘A in a von Neumann neighbourhood $\mathcal {N}_{\boldsymbol {r}}$Nr around node r (gray) followed by translocation $\mathcal {T}_{i}$Ti. (a) During reorientation $\mathcal {R}$R cells are stochastically redistributed within nodes according to a probability function P. The highest probability is assigned to a resulting node configuration η ′(r)with post-reorientation momentum J:=J(η ′,a ″)(r)parallel to the pre-reorientation local adhesivity gradient G:=G(η,a ′)(r)of the neighbourhood excluding r [Fig. 1b]. (b) In our model, the newly introduced adhesivity update operator $\mathcal {A}$A couples the time scales of the LGCA and ODE models. The graph shows an example for the [file 13062_2017_188_MOESM2_ESM.pdf]

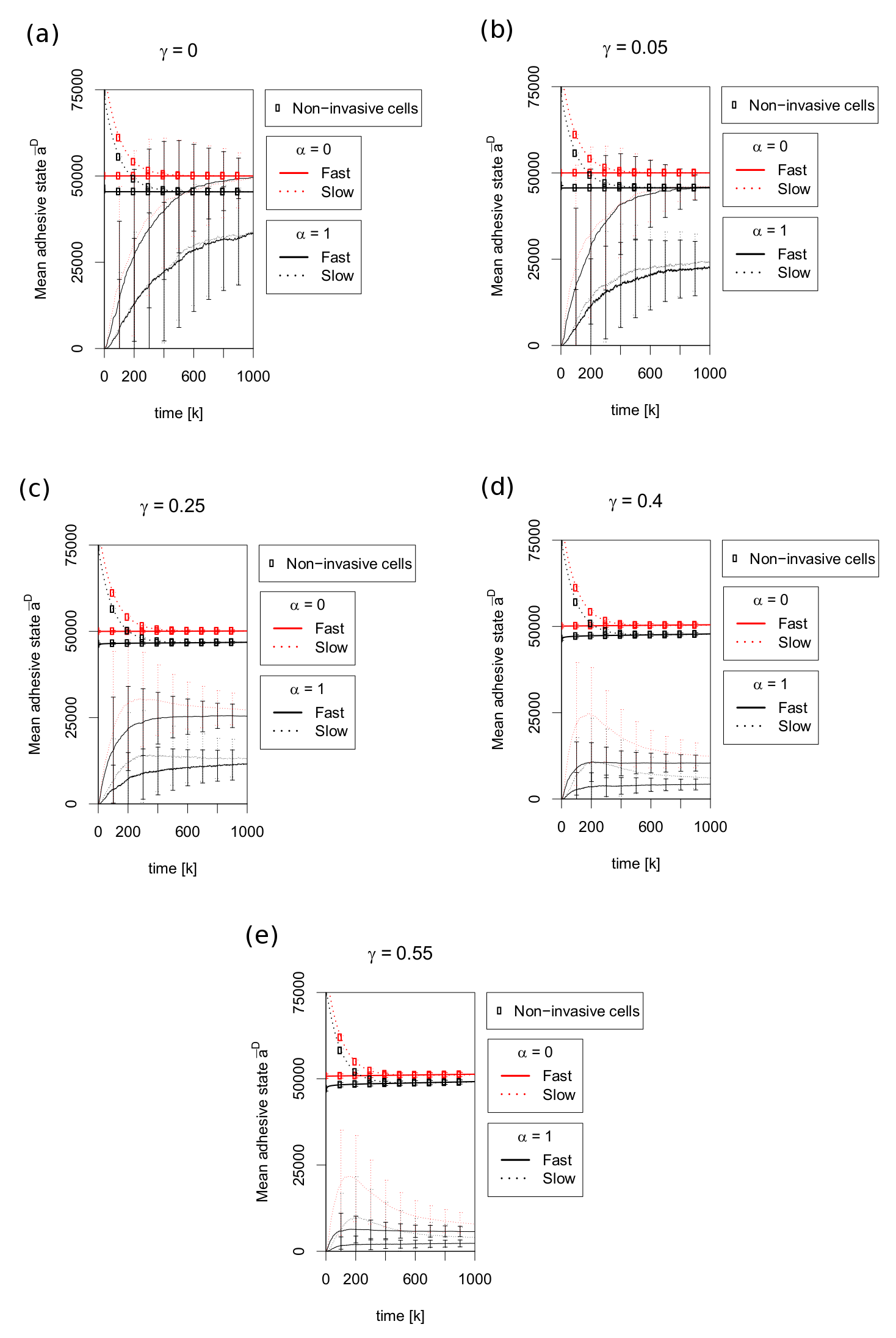

Supplement: Supplementary file 3 — Mean adhesive states comparison. Mean adhesive states comparison for disseminated cells in populations with different adhesion heterogeneity parameters γ and α=1. The plots show the mean adhesive states [Eq. (4)] of disseminated cells $\bar {a}^{D}$āD (curves) and non-disseminated cells $\bar {a}^{N}$āN (curves + circles) over time for γ=0,0.05,0.25,0.4,0.55 and α=1. Both $\bar {a}^{D}$āD and $\bar {a}^{N}$āN converge towards equilibrium states for all γ-values. The decrease of mean equilibrium adhesive states with increasing γ-values is significantly stronger between and for fixed γ in scenarios in which cellular adhesion phenotypes are under environmental control (red, Additional files 5 and 6). (PNG 347 kb) [file 13062_2017_188_MOESM3_ESM.png]

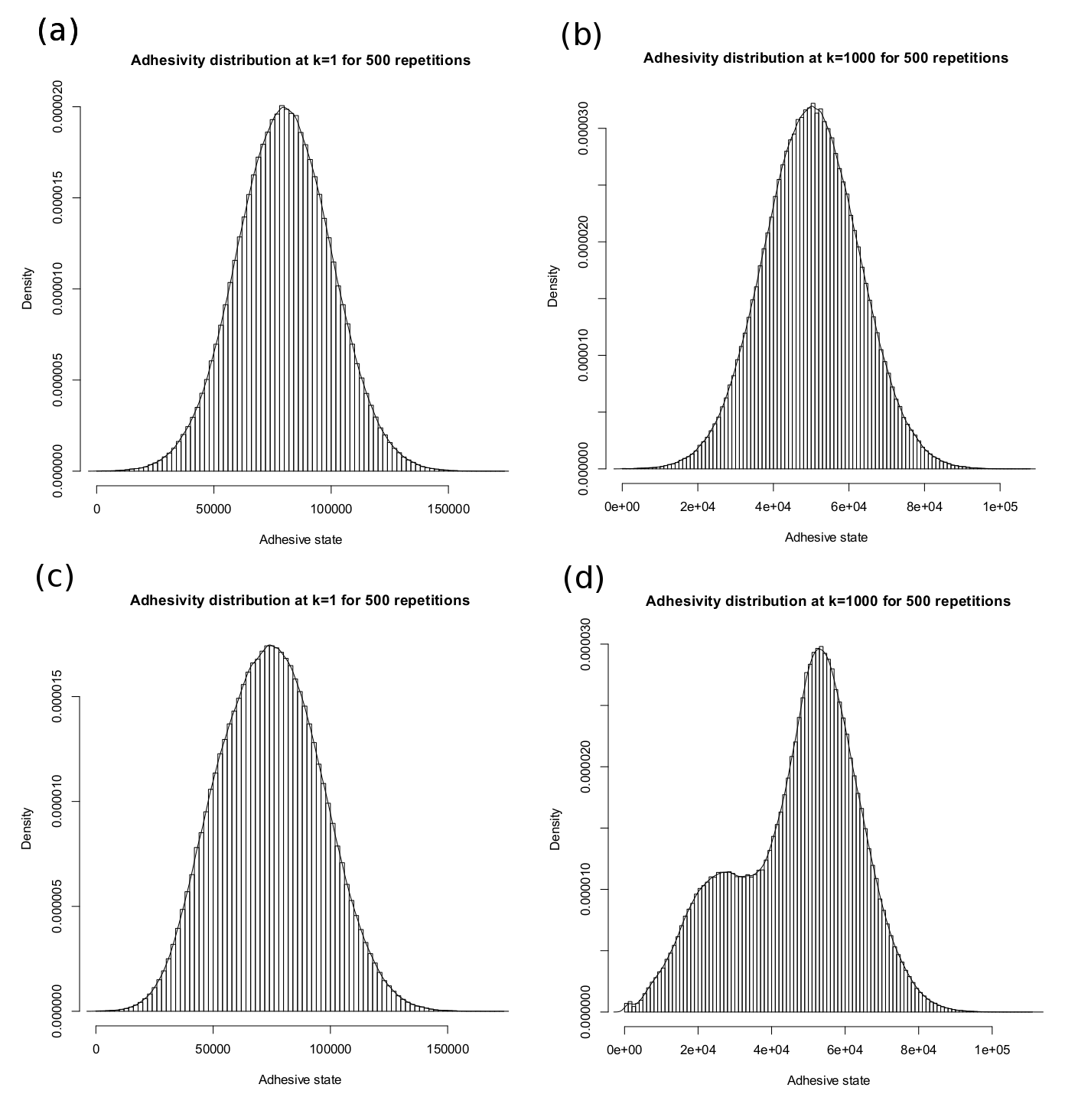

Supplement: Supplementary file 10 — Equilibrium mean adhesive state distributions. Equilibrium mean adhesive state distributions in cell populations with adhesion heterogeneity (γ=0.25) and slow regulation mode. (a) and (b) show the distributions of mean adhesive states $\bar {a}$ā in equilibrium for cell populations with only intrinsic adhesion heterogeneity and the slow regulation mode at time k=1 and k=1000 (Scenario I). (c) and (d) show the distributions of $\bar {a}$ā in equilibrium for cell populations with additional extrinsic adhesion heterogeneity and the slow regulation mode at time k=1 and k=1000 (Scenario III). The equilibrium adhesivity distributions do not differ when the fast regulation mode is considered (Fig. 8). (PNG 7966 kb) [file 13062_2017_188_MOESM10_ESM.png]

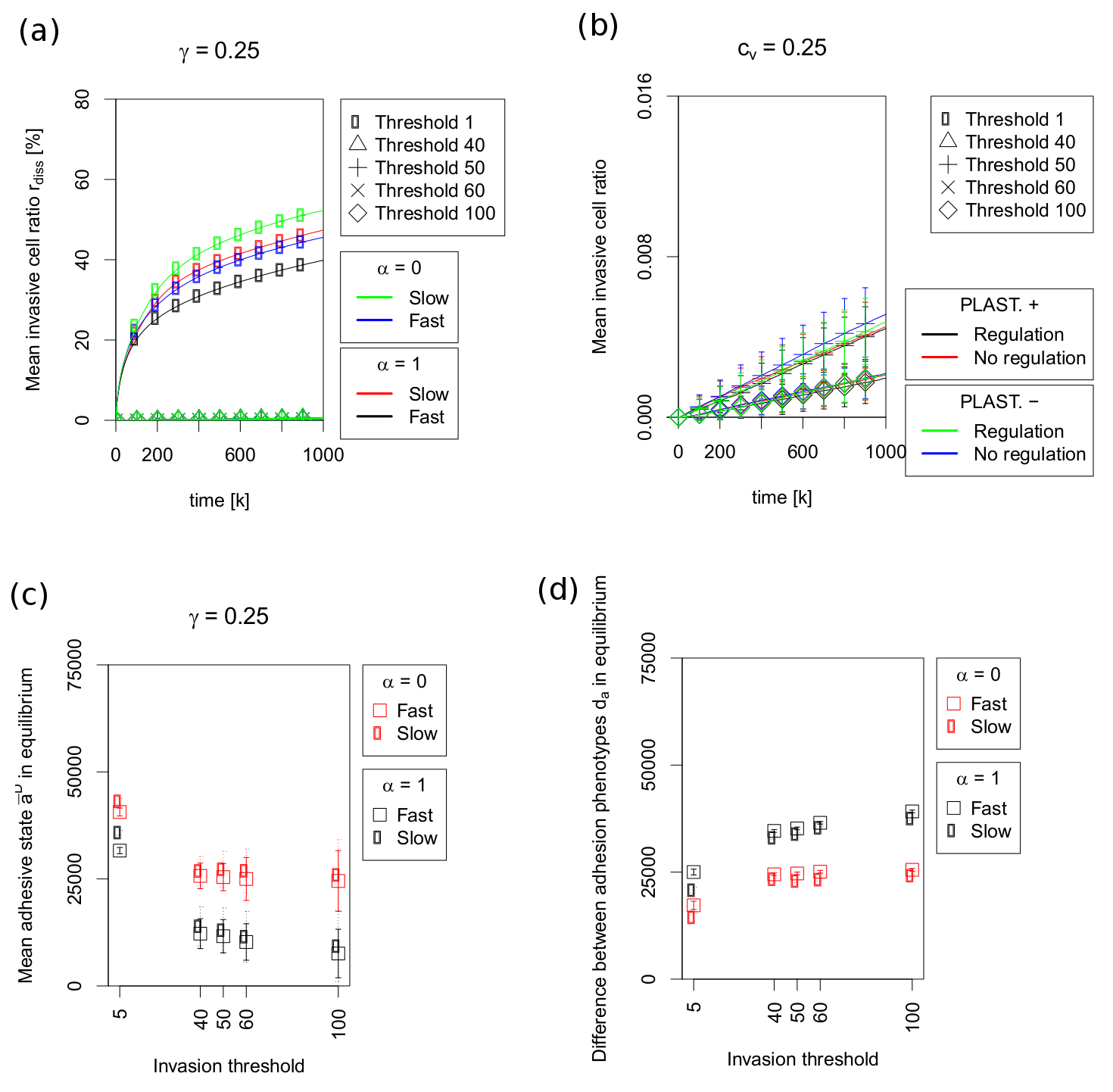

Supplement: Supplementary file 11 — Sensitivity to the cell dissemination threshold distanc. Sensitivity to the cell dissemination threshold distance for γ=0.25. (a) shows the disseminated cell ratio rdiss over time for different threshold distances. (b) shows rdiss over time for different threshold distances in a higher resolution for low values. From both (a) and (b) one can see that rdiss decreases with the cell dissemination threshold distance as would be expected. There is a striking difference for the cell dissemination threshold distance of 5 where rdiss increases drastically until it saturates at high ratios. (c) shows the mean equilibrium adhesive state of disseminated cells $\bar {a}^{D}$āD for different threshold distances. (d) shows the difference in adhesion phenotypes da between the two subpopulations for different threshold distances. As would be expected, the adhesion phenotype does not strongly depend on the distance threshold except for a very low distance threshold of 5. In the latter case more than half of the cells are considered disseminated so that the differentiation between the adhesion phenotypes is blurred. This is not surprising as within such short distance cells are likely to disseminate and re-join the cell population due to stochasticity. Accordingly, the effect is rather a model artefact than a biological phenomenon. (PNG 8294 kb) [file 13062_2017_188_MOESM11_ESM.png]

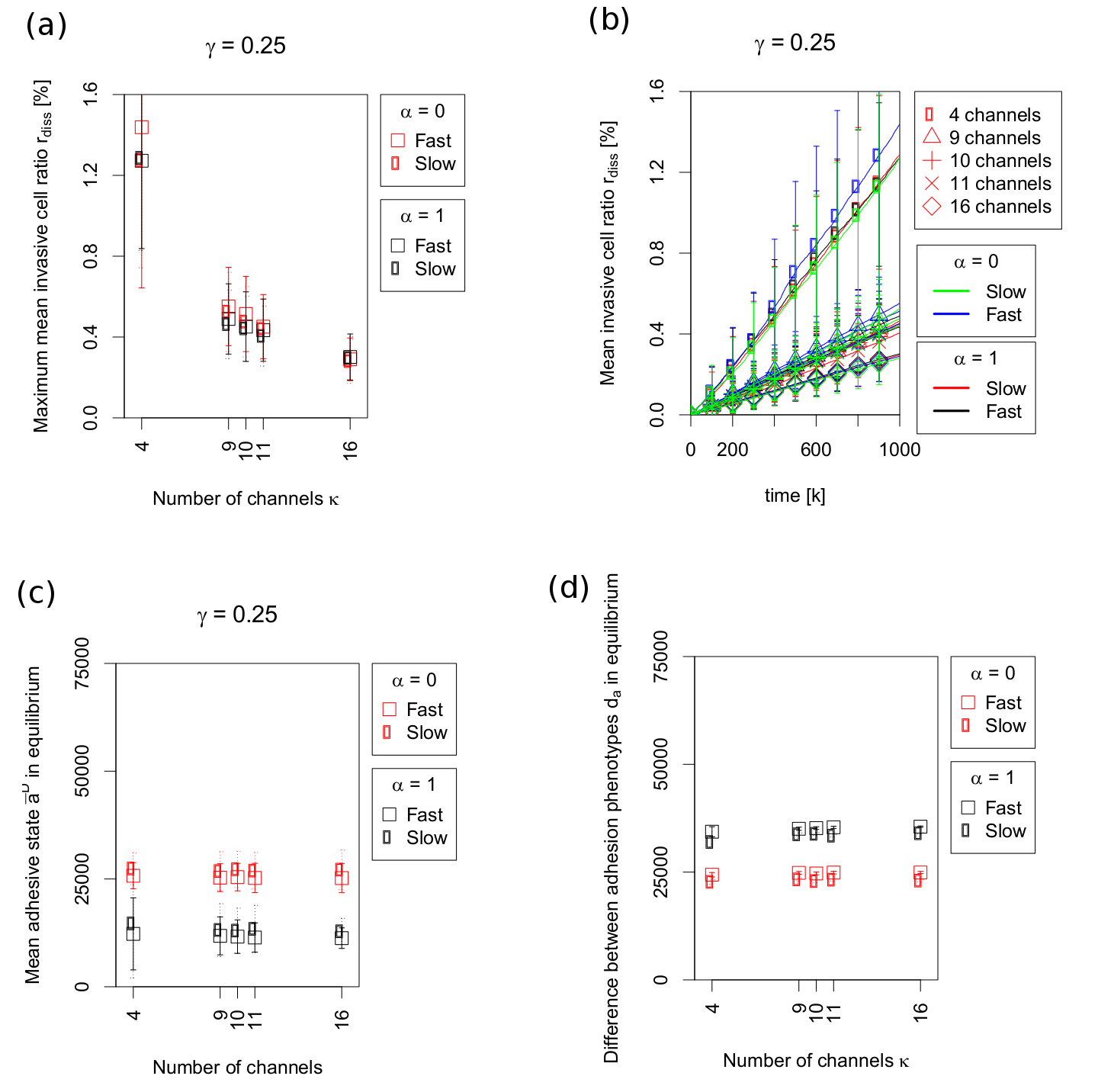

Supplement: Supplementary file 12 — Sensitivity to the number of channel. Sensitivity to the number of channels κ for γ=0.25. (a) shows the maximum value of the disseminated cell ratio rdiss for different values of κthat affect the number of rest channels β. (b) shows rdiss over time for different values of κ. Both the maximum of rdiss and the slope of rdiss as a function of time decrease with κ as expected. This is due to lower mobility caused by a lower numbers of rest channels. (c) shows the mean equilibrium adhesive state of disseminated cells $\bar {a}^{D}$āD for different values of κ. (d) shows the difference in adhesion phenotypes da between the two subpopulations for different values of κ. As expected, the adhesion phenotype does not depend on κ. (PNG 8058 kb) [file 13062_2017_188_MOESM12_ESM.png]

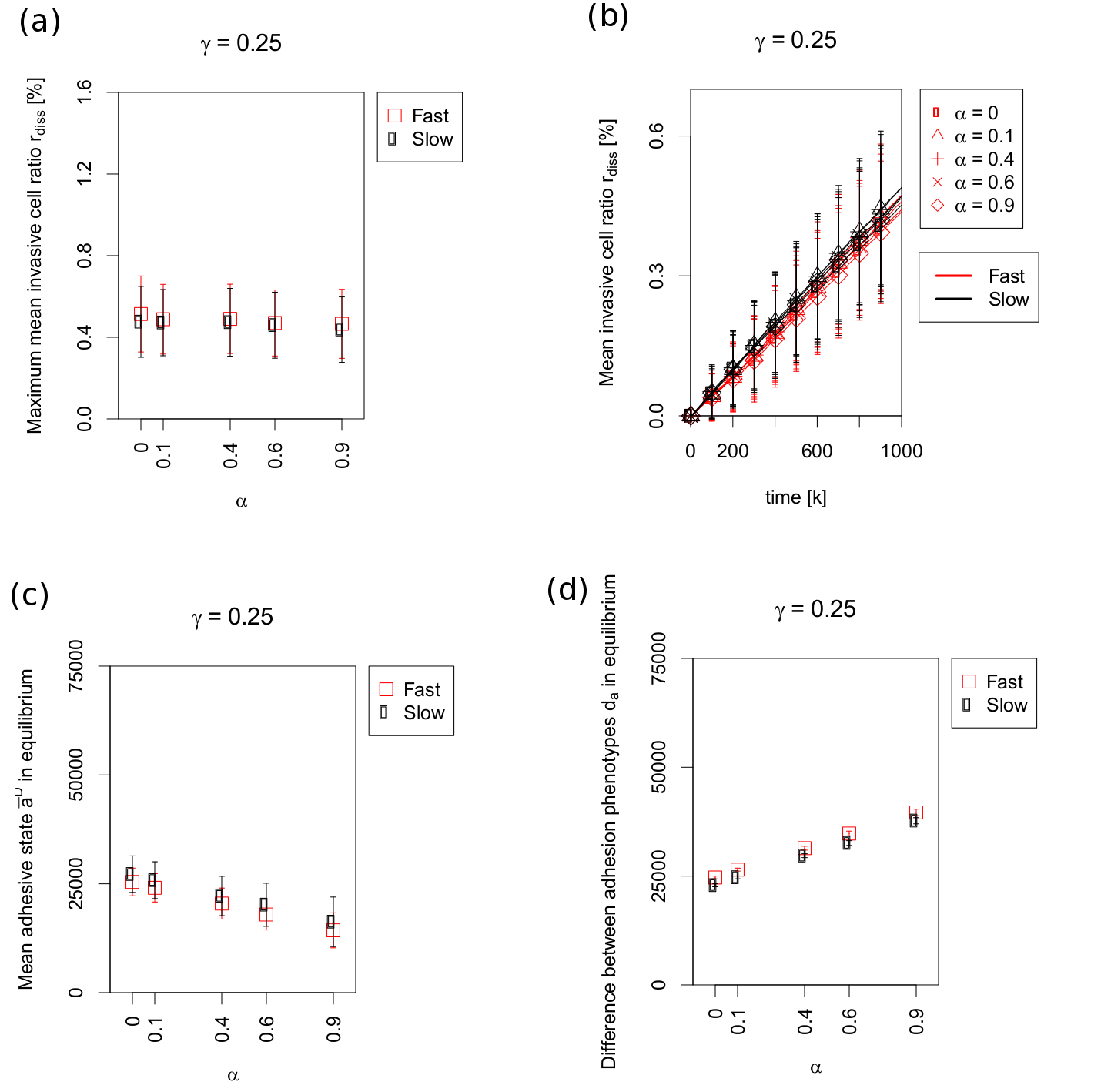

Supplement: Supplementary file 13 — Sensitivity to the environmental control parameter. Sensitivity to the environmental control parameter α for γ=0.25. (a) shows the maximum value of the disseminated cell ratio rdiss for different values of α. (b) shows rdiss over time for different values of α. Both the maximum of rdiss and rdiss over time do not depend on α. (c) shows the mean equilibrium adhesive state of disseminated cells $\bar {a}^{D}$āD for different values of α. (d) shows the difference in adhesion phenotypes da between the two subpopulations for different values of α. Whereas the mean equilibrium value of $\bar {a}^{D}$āD decreases with α, the distance da between the adhesion phenotypes increasesdue to growing influence of the environmental control mechanism (Fig. 7). (PNG 7925 kb) [file 13062_2017_188_MOESM13_ESM.png]
